# Characterization of *Dipsacus fullonum* L. Extract and Its Active Components as Natural Additives to Prevent Enzymatic Browning

**DOI:** 10.3390/plants15152374

**Published:** 2026-08-03

**Authors:** Oscar B. Micheloni, Mario O. Salazar, I. Ayelen Ramallo, Abel E. Farroni, Ricardo L. E. Furlan

**Affiliations:** 1 Departamento de Ciencias Básicas, Universidad Nacional del Noroeste de la Provincia de Buenos Aires, Pergamino B2700, Argentina; obmicheloni@comunidad.unnoba.edu.ar; 2 Area Farmacognosia, Facultad de Ciencias Bioquímicas y Farmacéuticas, Universidad Nacional de Rosario-CONICET, Rosario S2002LRK, Argentina; msalazar@fbioyf.unr.edu.ar (M.O.S.); aramallo@fbioyf.unr.edu.ar (I.A.R.); 3 Laboratorio de Biotecnología, Estación Experimental Agropecuaria Pergamino, Instituto Nacional de Tecnología Agropecuaria, Pergamino CC 31-B2700WAA, Argentina

**Keywords:** polyphenol oxidase, natural food additives, bioautography, enzymatic browning, iridoid glycosides, sylvestrosides, fresh-cut produce

## Abstract

This study addresses the need for effective natural inhibitors of enzymatic browning in fresh-cut produce by exploring plant-derived compounds with activity against apple polyphenol oxidase (PPO). Extracts from 26 wild plant species were screened using thin-layer chromatography (TLC)-coupled bioautographic assays specifically adapted to apple PPO. Among these, *D. fullonum* showed clear inhibitory activity and was selected for further investigation. Extracts from different plant organs revealed that PPO inhibition was localized in the leaves, while stems and flower receptacles were inactive. Bioautography-guided fractionation of leaf extracts enabled the isolation of active fractions, and structural characterization by HRMS and NMR indicated that the activity is associated with the iridoid glycosides sylvestrosides **III** and **IV**. Antioxidant assays (DPPH, ABTS, CUPRAC) and TLC profiling demonstrated that PPO inhibition was not directly correlated with radical scavenging activity. In situ application of the leaf extract (0.5% *w*/*v*) on fresh-cut apple slices significantly delayed browning during refrigerated storage, showing comparable or improved performance relative to ascorbic acid at later stages. These results highlight *D. fullonum* as a promising source of natural antibrowning agents and demonstrate the utility of food-specific bioautographic assays for the targeted discovery of functional plant metabolites.

## 1. Introduction

The demand for fresh-cut fruits and vegetables has increased in recent years due to consumer preference for convenient and minimally processed foods. However, tissue disruption during processing induces physiological responses in plant tissues that negatively affect quality. Among these, enzymatic browning is one of the most relevant, as it alters visual appearance and reduces shelf life [1]. This process is mainly catalyzed by polyphenol oxidases (PPOs), copper-containing enzymes widely distributed in plants that oxidize phenolic compounds to quinones, which subsequently polymerize into brown pigments [2]. PPOs exhibit catechol oxidase (CO) and tyrosinase (TYR) activities, differing in their catalytic properties. Importantly, PPOs show significant variability among plant species, tissues, and developmental stages, which influences their substrate specificity and sensitivity to inhibitors [3].

Chemical control of enzymatic browning is based on compounds that interfere with PPO activity or with associated oxidative reactions [4]. However, screening for PPO inhibitors is commonly performed using commercial mushroom tyrosinase, which does not adequately represent the diversity of plant PPOs. As a result, compounds identified as active in these assays may show limited effectiveness when applied to specific plant tissues, where PPO isoforms differ in their biochemical properties [5]. This limitation highlights the need for screening strategies based on enzyme sources relevant to the target plant system.

Conventional antibrowning agents, such as sulphites, ascorbic acid, cysteine, and kojic acid, present limitations including safety concerns, transient effects, undesirable sensory properties, or high production costs [4,6]. Therefore, there is increasing interest in identifying natural inhibitors derived from plants. Despite extensive research, few natural compounds have demonstrated sufficient efficacy and applicability, emphasizing the need for improved discovery approaches.

Wild plant species represent a valuable source of bioactive metabolites, particularly those traditionally used as food or medicine [7,8]. However, the identification of bioactive metabolites as bioactivity markers within complex mixtures of compounds still represents a significant challenge. Plant-derived PPO inhibitors encompass a wide range of natural compounds, including phenolic compounds, carotenoids, organic acids, and terpenoids [9]. Among these, phenolic compounds (e.g., phenolic acids [10,11,12], flavonoids [9,13,14,15], stilbenes [16,17,18], and phenolic-rich extracts [19,20,21] are the most extensively studied. Their antibrowning activity has been attributed to multiple mechanisms, including direct inhibition of PPO through interactions with the enzyme active site and copper chelation, as well as antioxidant activity that reduces quinone accumulation and the subsequent formation of brown pigments [22,23]. Organic acids have also been reported to reduce enzymatic browning through pH modulation and copper chelation [24], whereas carotenoids and terpenoids have shown promising antibrowning activity, although these chemical classes remain comparatively less explored [22,23]. Despite the increasing interest in plant-derived PPO inhibitors, the identification of more effective compounds with suitable application properties remains necessary, as some plant extracts require relatively high concentrations to achieve sufficient activity or may impart undesirable sensory characteristics [25].

Thin-layer chromatography (TLC)-coupled bioautographic methods enable the separation of complex extracts and the direct detection of biological activity on the chromatographic plate, providing an efficient tool for the identification of active compounds such as enzyme inhibitors [26,27]. These methods can be adapted to use enzyme extracts obtained from specific plant tissues, allowing the identification of inhibitors with relevance to the biological system under study [28,29].

Although the phytochemical composition of *D. fullonum*, including iridoids, phenolic acids and flavonoids, has been previously investigated [30,31,32,33,34], its potential as a source of inhibitors of food-relevant polyphenol oxidases has not been explored. Moreover, no studies have combined bioactivity-guided chromatographic fractionation with direct evaluation of antibrowning performance in fresh-cut produce. In this context, the present study aimed to apply a TLC-based bioautographic assay using apple-derived PPO to screen extracts from wild plant species, identify the metabolites responsible for the inhibitory activity, and evaluate their practical effectiveness in reducing enzymatic browning in fresh-cut apples. This approach links phytochemical characterization with functional validation, providing a workflow for the discovery of natural antibrowning agents with potential applications in minimally processed foods.

## 2. Results and Discussion

### 2.1. Screening of Extracts for PPO Inhibition

To identify potential natural inhibitors of polyphenol oxidase (PPO), crude extracts from twenty-six species belonging to twenty-six different genera, selected from plants traditionally used in folk medicine or as foods [35,36,37,38,39,40,41,42,43] were evaluated. Forty-seven crude extracts were obtained using different extraction procedures.

**Figure 1:**
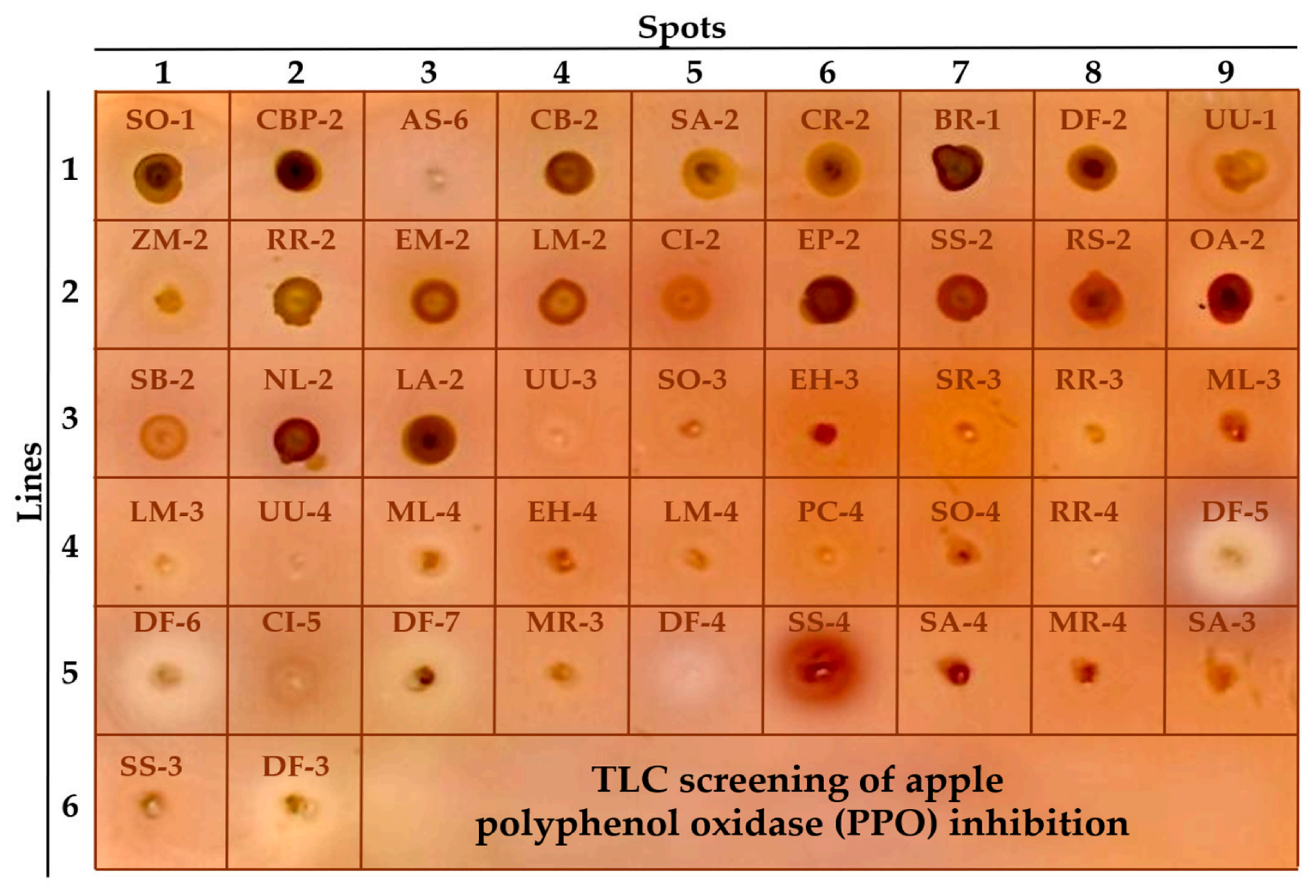
TLC plates visualized evaluated using the PPO–TLC assay for the screening of extracts (400 µg/spot). Each sample was coded according to the plant species initials and the extraction protocol employed: reflux with 96% ethanol (1), reflux with methanol (2), citrate buffer extraction (40 °C, pH 4.5) (3), enzyme-assisted extraction using Viscozyme in citrate buffer (40 °C, pH 4.5) (4), infusion (5), decoction (6), and maceration (7). **Line 1 spot 1–9:** *Sonchus. oleraceus* L. (SO-1), *Capsella bursa-pastoris* (L.) Medik. (CBP-2), *Alium. sativum* L. (AS-6), *Cardamine bonariensis* Pers. Var. *bonariensis* (CB-2), *Sinapis arvensis* L. (SA-2), *Cerastium rivulariastrum* Moeschi & Pedersen (CR-2), *Brassica rapa* L. (BR-1), *D. fullonum* (DF-2), and *Urtica urens* L. (UU-1). **Line 2 spot 1–9:** *Zea mays* (ZM-2), *R. rugosum* (RR-2), *Erodium malacoides* L. (EM-2), *Lolium multiflorum* Lam. (LM-2), *Cichorium intybus L.* (CI-2), *Echium plantagineum* L. (EP-2), *Solanum sisymbriifolium* Lam. Var. *sisymbriifolium* (SS-2), *Raphanus sativus* L. (RS-2), and *O. articulata* (OA-2). **Line 3 spot 1–9:** *Sphaeralcea bonariensis* (Cav.) Griseb. (SB-2), *Nicotiana longiflora* Carv. (NL-2), *Lamium amplexicaule* L. (LA-2), *U. urens* (UU-3), *S. oleraceus* (SO-3), *Eryngium horridum* Malme (EH-3), *Sida rhombifolia* L. (SR-3), *R. rugosum* (RR-3), and *M. lupulina* (ML-3). **Line 4 spot 1–9:** *L. multiflorum* (LM-3), *U. urens* (UU-4), *M. lupulina* (ML-4), *E. horridum* (EH-4), *L. multiflorum* (LM-4), *Passiflora caerulea* L. (PC-4), *S. oleraceus* (SO-4), *R. rugosum* (RR-4), and *D. fullonum* (DF-5). **Line 5 spot 1–9:** *D. fullonum* (DF-6), *C. intybus* (CI-5), *D. fullonum* (DF-7), *Matricaria recutita* L. (MR-3), *D. fullonum* (DF-4), *S. sisymbriifolium* (SS-4), *S. arvensis* (SA-4), *M. recutita* (MR-4), and *S. arvensis* (SA-3). **Line 6 spot 1–9:** *S. sisymbriifolium* (SS-3), and *D. fullonum* (DF-3).

All extracts were evaluated for PPO inhibitory activity using a TLC-PPO assay at 400 µg/spot (Figure 1), revealing marked differences depending on both plant source and extraction protocol. While moderate activity was observed for the methanol reflux extract from *Oxalis articulata* Savigny (OA-2, Figure 1, line 2, spot 9), the citrate buffer extract of *Rapistrum rugosum* L. (RR-3, Figure 1, line 3, spot 8), and the Viscozyme assisted extract of *Medicago lupulina* L. (ML-4, Figure 1, line 4, spot 3), the highest inhibitory activity was observed for the aqueous extracts of *D. fullonum* obtained by infusion and decoction (DF-5 and DF-6, Figure 1, line 4, spot 9 and line 5, spot 1, respectively). The methanolic extract of *D. fullonum* exhibited no detectable activity (DF-2, Figure 1, line 1, spot 8), possibly due to poor extraction selectivity for the active compounds or their degradation. In addition, the Viscozyme-assisted extract (DF-4, Figure 1, line 5, spot 5) showed reduced activity, which may be attributed to the release of non-active matrix components following enzymatic cell wall degradation, leading to dilution or masking of the active constituents in the crude extract. Finally, the maceration extract (DF-7, Figure 1, line 5, spot 3) and the citrate buffer extract prepared at 40 °C (DF-3, Figure 1, line 6, spot 2), exhibited lower PPO-inhibitory activity than the extracts obtained at higher temperatures, such as DF-6 and DF-5.

**Figure 2:**
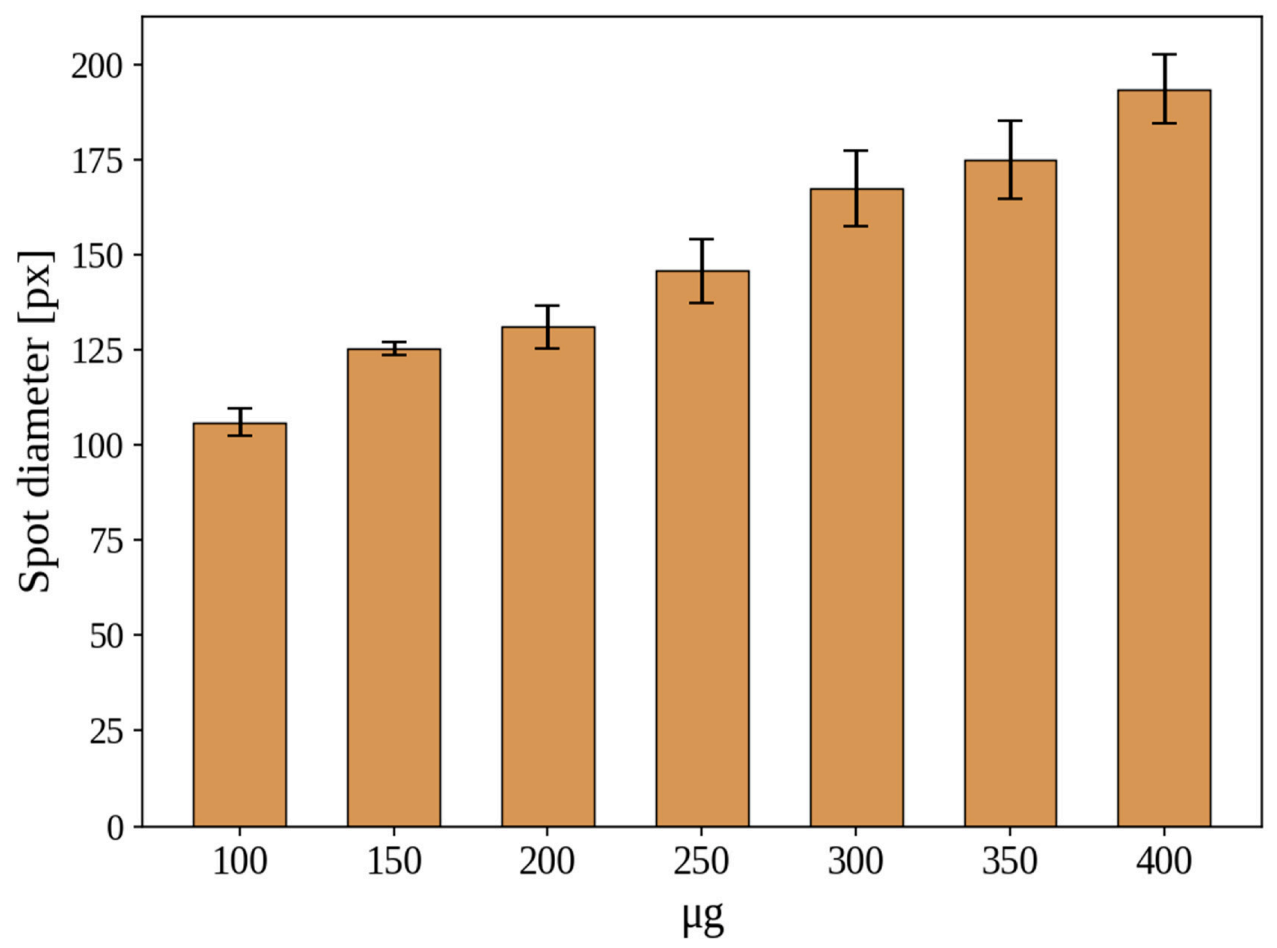
Dose–response analysis of the DF-6 extract. TLC plates were spotted with increasing amounts of the DF-6 extract (100–400 µg) and evaluated using the PPO–TLC assay. Bars represent the mean halo diameter ± SD (*n* = 12 diametral measurements per spot, taken at 15° intervals through the spot centroid on ImageJ 1.53c-binarized images).

The extract of *D. fullonum* obtained by aqueous decoction (DF-6) was selected to assess PPO inhibition as a function of loading (Figure 2). TLC plates were spotted with increasing amounts of DF-6 extract (100–400 µg/spot) and evaluated using the PPO–TLC assay. The diameter of the resulting inhibition halos increased consistently with extract loading, from 106.0 ± 3.6 px at 100 µg to 193.5 ± 9.1 px at 400 µg (mean ± SD of twelve diametral measurements per spot; Table S1). This behaviour indicates a clear dose-dependent inhibitory effect, consistent with the presence of active constituents responsible for PPO inhibition.

**Figure 3:**
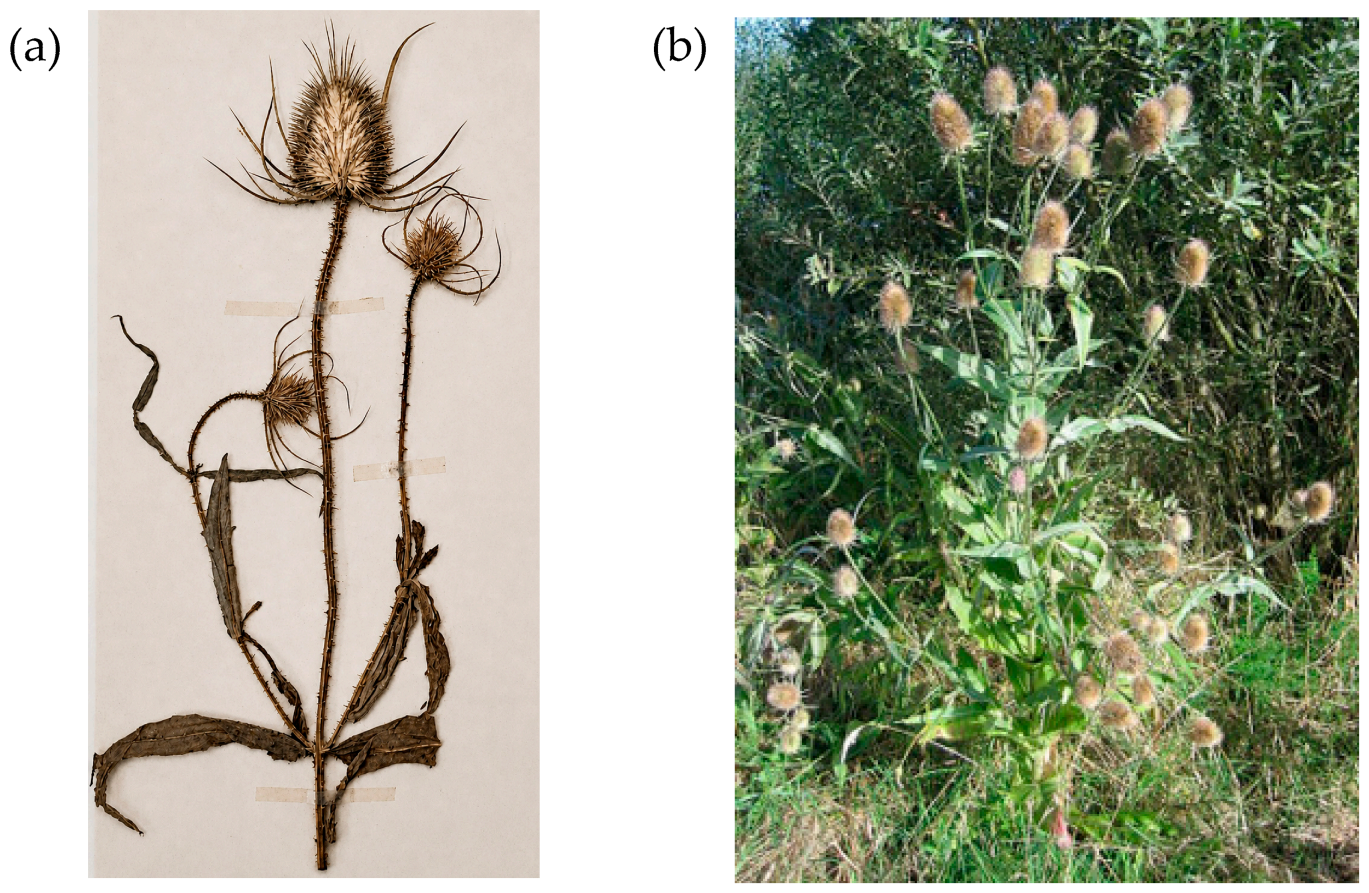
(**a**) Voucher specimen of *D. fullonum*, originally collected and currently deposited in the university herbarium. (**b**) Representative photograph of *D. fullonum* in its vegetative stage. Photograph by MPF (Wikimedia Commons), reproduced under the Creative Commons Attribution-ShareAlike 3.0 (CC BY-SA 3.0) licence. Available at: https://commons.wikimedia.org/wiki/File:Dipsacus_fullonum1.jpg. Accessed on 22 July 2026.

*D. fullonum* (Dipsacaceae) is a widely distributed biennial species (Figure 3), considered invasive in several regions [44], and is also present in Argentina, where it has been used in traditional preparations and ethnomedicine [37,38].

Previous studies have shown that this species contains diverse secondary metabolites, including chlorogenic acid derivatives, flavonoids, and iridoid glycosides [32,45]; therefore, the bioactive DF extracts likely comprise a complex mixture of compounds. Consequently, the observed PPO inhibition may result from a single potent inhibitor, multiple mild inhibitors, or an intermediate scenario involving compounds with different contributions to the overall activity. Distinguishing between these possible scenarios is important, as it enables more informed decision-making during the early stages of the research process.

**Figure 4:**
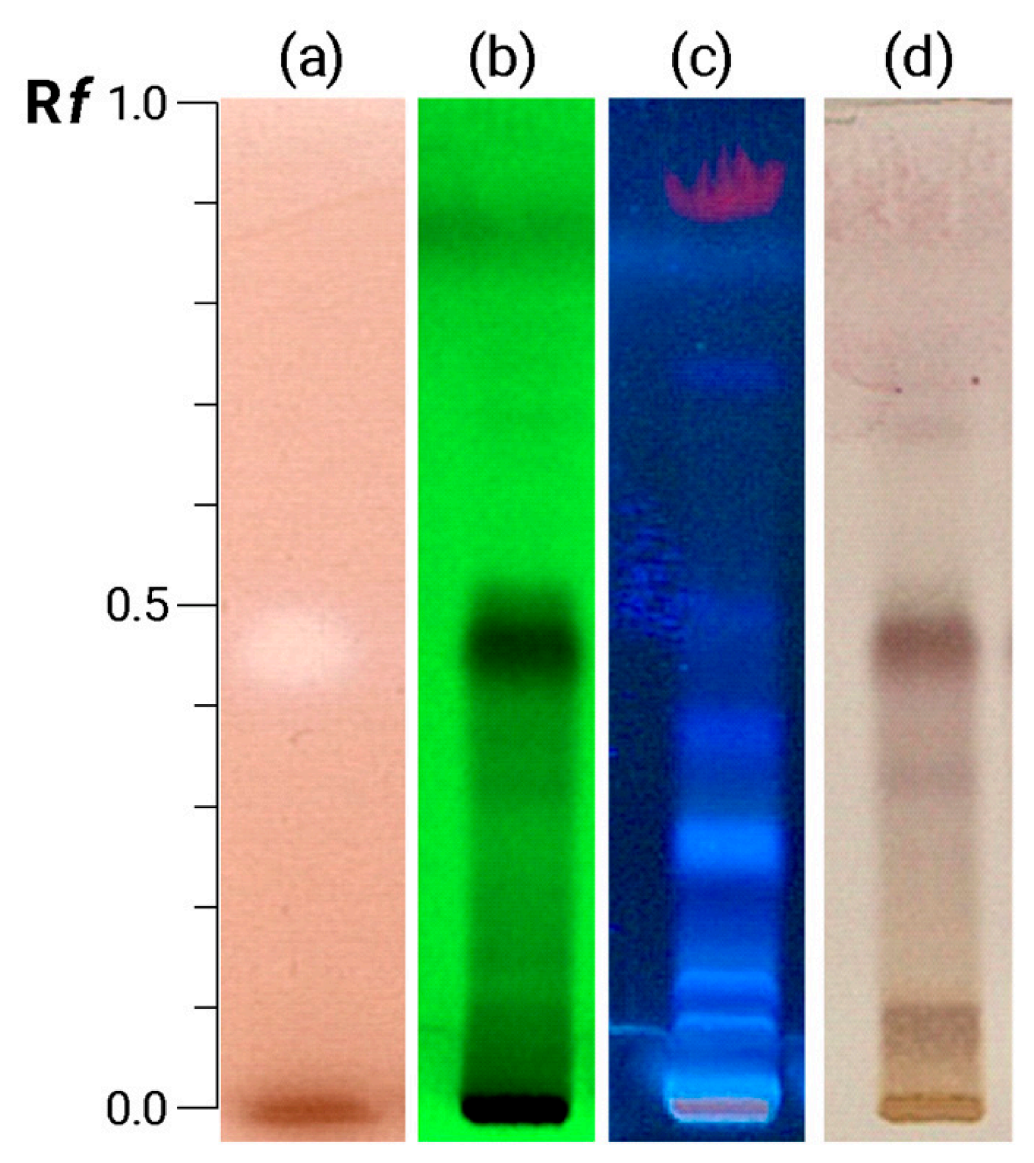
TLC chromatograms of DF-6 extract (400 µg) visualized under different conditions: PPO–TLC assay (**a**), UV light at 254 nm (**b**), UV light at 365 nm (**c**), and vanillin–sulphuric acid reagent (**d**). Mobile phase consisted of chloroform:methanol:water (85:25:1).

One advantage of using inhibition assays on TLC plates is that bioactive mixtures can be separated before assessing PPO activity in situ. By adding a chromatographic dimension, this approach provides preliminary insight into the components responsible for the inhibitory properties of the mixture using only microgram quantities of sample. Accordingly, the developed chromatogram of the DF-6 extract was evaluated in situ for PPO activity. A single inhibition zone with an *Rf* value of 0.45 was observed (Figure 4a). Although this spot was not visible when the plate was examined under 365 nm UV light (Figure 4c), the inhibition zone coincided with spots at the same *Rf* value detected under 254 nm UV light and after derivatization with vanillin–sulphuric acid reagent (Figure 4b,d). These observations indicate that, although the extract contains numerous components, the PPO inhibition is mainly attributable to either a single compound or a group of compounds with similar chromatographic behaviour under these conditions. The PPO inhibition by the DF-6 extract was also quantified in a microplate assay, yielding an IC_50_ value of 2.7 ± 0.5 mg/mL and confirming the inhibition previously observed on the PPO–TLC assay.

**Figure 5:**
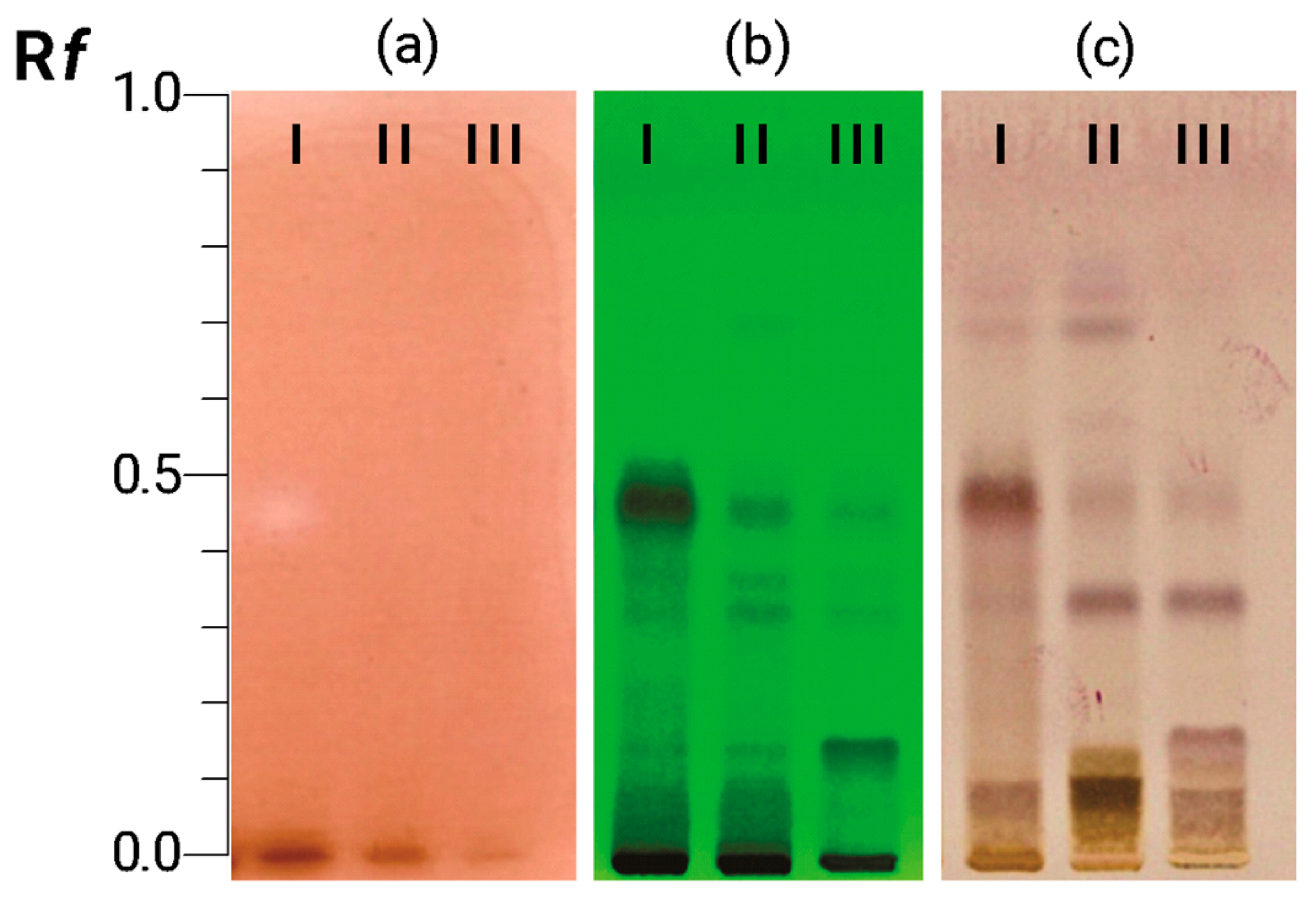
TLC chromatograms of DF sub-extracts (400 µg) DF_L_6 (track I), DF_F_-6 (track II), and DF_S_-6 (track III), visualized under different conditions: (**a**) PPO–TLC assay; (**b**) UV light at 254 nm; (**c**) vanillin–sulphuric acid reagent. Mobile phase consisted of chloroform:methanol:water (85:25:1).

Since the active DF-6 extract was prepared from vegetal material comprising different aerial parts (stems, leaves, and flower receptacles), three additional extracts were prepared to further characterize its activity. Each extract was obtained from a single type of aerial part: stems (DF_S_-6), leaves (DF_L_-6), and flower receptacles (DF_F_-6). When analyzed with the PPO–TLC assay, only the DF_L_-6 extract (Figure 5a, track I) showed an inhibition halo with an *Rf* value of 0.45, suggesting that it contains the PPO inhibitor(s) previously detected in the aerial parts DF-6 extract. This inhibition zone coincided with spots at the same *Rf* value observed under UV light at 254 nm (Figure 5b, track I) and under white light after derivatization with vanillin–sulphuric acid reagent (Figure 5c, track I). In contrast, DF_S_-6 and DF_F_-6 (Figure 5a, tracks II and III) did not produce any inhibition zones, likely because the concentration of inhibitory constituents in stems and flower receptacles is too low to be detected. This is supported by the profiles observed under UV light at 254 nm (Figure 5b) and under white light after derivatization with vanillin–sulphuric acid reagent (Figure 5c).

### 2.2. Total Phenolic Compounds and Antioxidant Activity of DF Extracts

The antioxidant capacity and total phenolics of DF-6 extracts and sub-extracts derived from different aerial parts were determined using DPPH, ABTS^•+^, and CUPRAC assays. The IC_50_ values, reduction capacity, and total phenolics are summarized in Table 1. Ascorbic acid (AA) and Trolox^®^ (TL) were included as standards to compare scavenging activity but excluded from the statistical analysis to allow comparison among plant extracts.

Statistical analysis (ANOVA) revealed significant differences among the extracts depending on the assay (*p* < 0.05), as indicated by the different letters in Table 1. In the DPPH assay, DF-6, DF_L_-6, and DF_F_-6 did not differ significantly, whereas DF_S_-6 showed significantly lower activity. In contrast, the ABTS assay revealed significant differences among all extracts, with DF_L_-6 exhibiting the highest ABTS scavenging capacity. For CUPRAC, DF-6 exhibited the highest reducing capacity, followed by DF_L_-6 and DF_F_-6, which did not differ significantly from each other, while DF_S_-6 showed the lowest activity. Total phenolic content also differed significantly among all extracts, increasing in the order DF_S_-6 < DF-6 < DF_F_-6 < DF_L_-6.

Correlation analysis was performed to explore relationships among the evaluated antioxidant parameters. Although some associations were observed, the limited number of samples (*n* = 4) restricts the statistical robustness of the results; therefore, the correlations should be considered indicative rather than conclusive. Spearman analysis revealed a strong negative association between total phenolic content and both DPPH scavenging activity (ρ ≈ −0.90) and ABTS^•+^ scavenging activity (ρ = −1.00), indicating that phenolic compounds are major contributors to radical scavenging capacity in these extracts. In contrast, only a moderate positive correlation was observed with CUPRAC (ρ ≈ 0.45), suggesting the involvement of additional compounds and/or different reaction mechanisms. Overall, these findings suggest that antioxidant activity may depend not only on total phenolic content, but also on the presence of specific compounds and their uneven distribution among plant organs. Differences among assays further highlight the influence of the underlying reaction mechanisms on the measured antioxidant activity.

Previous studies have reported the presence of several bioactive phenolics in *Dipsacus* species, including protocatechuic acid, which exhibits antioxidant and antidiabetic activities [46]. Oszmiański et al. reported higher polyphenol content in DF leaves than in roots and identified multiple phenolic acid derivatives and flavones, including apigenin and luteolin derivatives [33]. Other reports have also described the occurrence of compounds such as caffeic acid, vanillic acid, 2,6-dihydroxycinnamic acid, and caffeoylquinic acid in roots of *D. asper* and dipsaicin in aerial parts of *D. laciniatus* [47].

To further investigate whether the compounds responsible for PPO inhibition were the same as those contributing to antioxidant activity, the extracts were evaluated using DPPH, ABTS^•+^, and CUPRAC-TLC assays (Figure S1). The results indicate that different compounds are involved in these activities, as they exhibit distinct chromatographic behaviours. In all cases, multiple compounds appear to contribute to antioxidant activity, particularly on the ABTS^•+^ assay, where inhibition was observed at *Rf* values between 0 and 0.4. For DPPH and CUPRAC, stronger effects were observed at lower *Rf* values. Notably, no activity was detected at the *Rf* value of 0.45, corresponding to the PPO inhibition band, indicating that the compounds responsible for PPO inhibition differ from those contributing to the antioxidant capacity.

These findings suggest that PPO inhibition in DF extracts is not directly associated with radical scavenging mechanisms, pointing instead to alternative modes of enzyme inhibition.

### 2.3. Purification, Further Inhibition Studies, and Structural Characterization

As previously shown, an initial screening of aerial parts extracts identified the decoction and infusion extracts as the most active against PPO. Subsequent organ-specific extraction revealed that the activity was mainly associated with the leaf extract (DF_L_-6). Based on these findings, and to improve the selective extraction of active compound(s) over other constituents for subsequent cleaner bioguided fractionation and identification, additional extraction methods were evaluated using *D. fullonum* leaves. Accordingly, leaf extracts were prepared by sonication (DF_L_-8) and infusion (DF_L_-5), in addition to the previously tested decoction protocol.

**Figure 6:**
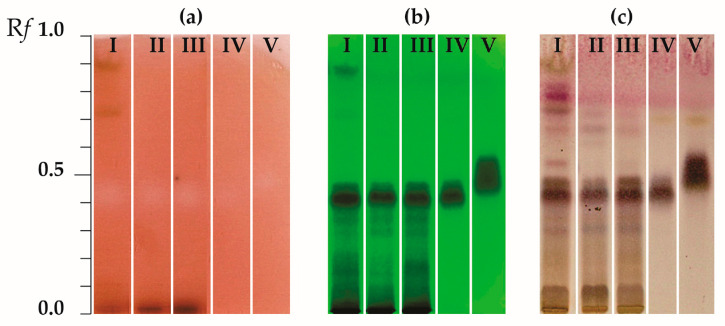
TLC chromatograms of DF extracts and fractions: DF_L_-8 (400 µg, track I), DF_L_-6 (400 µg, track II), DF_L_-5 infusion extract (400 µg, track III), A2I (50 µg, track IV), and A2II (50 µg, track V). Plates were visualized using: (**a**) PPO–TLC autographic assay; (**b**) UV light 254 nm; and (**c**) vanillin–sulphuric reagent. The mobile phase consisted of chloroform:methanol:water (85:25:1).

The extracts were compared using the PPO–TLC assay, UV light at 254 nm, and vanillin–sulphuric acid reagent (Figure 6). The PPO–TLC assay revealed the presence of the compound(s) responsible for PPO inhibition at an *Rf* of 0.45 in all three extracts (DF_L_-6, DF_L_-8, DF_L_-5, with comparable intensity (Figure 6a, tracks I–III). When visualized under UV light at 254 nm (Figure 6b) or after derivatization with vanillin–sulphuric acid reagent (Figure 6c), the extracts obtained by decoction and infusion showed similar chromatographic profiles (tracks II and III). In contrast, the sonication extract exhibited additional, less-polar compounds that were absent from the other extracts (track I). Since the infusion method provided the highest extraction yield (30%) while maintaining a similar chemical profile and PPO inhibitory activity, this extract was selected for further fractionation.

A PPO autography–guided column chromatography of the DF_L_-5 extract led to the isolation of two active fractions, A2I and A2II. Fraction A2I showed an inhibition halo with an *Rf* value of 0.45 (Figure 6a, track IV), while A2II showed an inhibition zone at an *Rf* value of 0.48 (Figure 6a, track V). These compounds were detected under UV light at 254 nm and after sulphuric–vanillin derivatization (Figure 6b,c, tracks IV and V). This reagent, although considered universal, is commonly used for the detection of terpenes, which react with vanillin to produce a violet-blue coloration [48]. Thus, these results suggest the presence of oxygenated terpenes, such as iridoids.

Different iridoid compounds have been identified in *Dipsacus* species. DF leaves and roots contain loganic acid, loganin, sweroside, cantleyoside, and sylvestrosides I-IV, and iridoid content is considered one of the main indicators of biological activity in this species [33]. Loganin and loganic acid have inhibitory effects on mushroom tyrosinase, although weaker than that of kojic acid. Sweroside showed capacity to inhibit body pigmentation and tyrosinase activity in a zebrafish in vivo model [49]. Although the phytochemical composition of *D. fullonum* has been investigated since the late 1970s [30], no studies have evaluated its constituents as inhibitors of apple-derived PPO or explored their potential to prevent enzymatic browning in foods. Therefore, the association of PPO inhibitory activity with fractions enriched in sylvestrosides **III** and **IV**, together with the subsequent demonstration of antibrowning activity in fresh-cut apples, represents the principal novelty of the present study.

**Figure 7:**
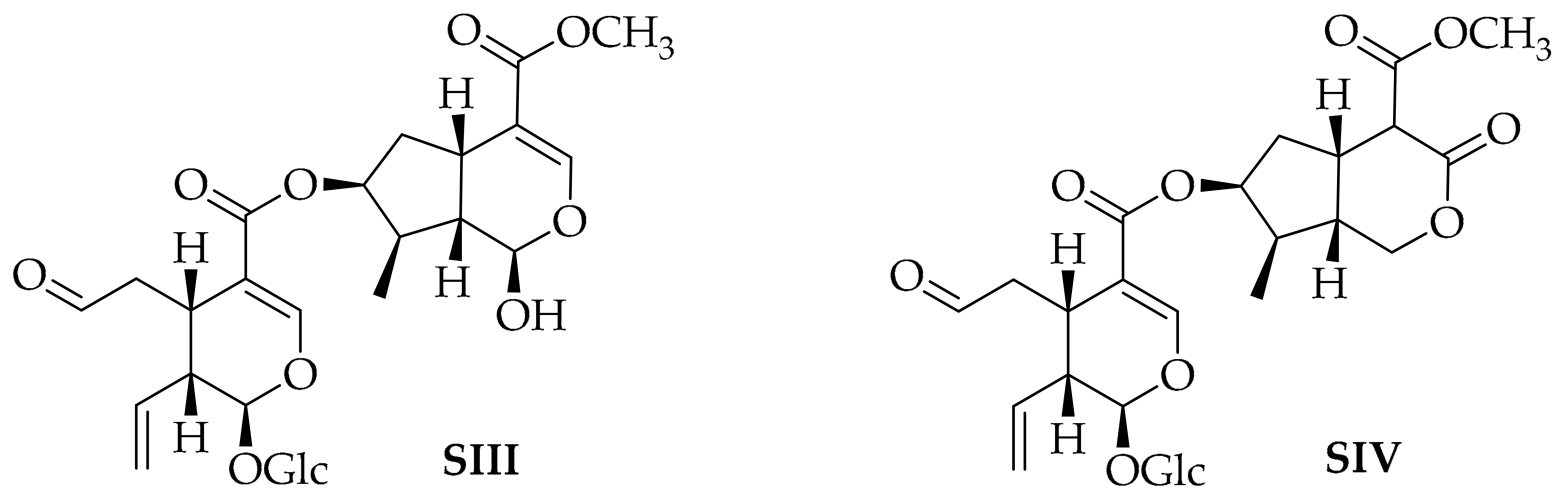
Chemical structures of sylvestrosides **III** (**SIII**) and **IV** (**SIV**).

High resolution mass spectrometry (HRMS) analysis of the fraction A2I in negative ionization mode (Figure S2a) provided a spectrum with two dominant signals at *m*/*z* 583.2047 (Figure S2b) and *m*/*z* 615.2308 (Figure S2c), which could correspond to deprotonated molecular ions associated with the molecular formulas C_27_H_36_O_14_ and C_28_H_40_O_15_, respectively (errors: 2.5 mDa and 2.5 mDa, respectively). These molecular formulas were confirmed in positive ionization mode; the sodium molecular ions at *m*/*z* 607.1985 and 639.2246 were the dominant signals in this case (Figure S2d). Comparison with published data of known iridoids showed the molecular formula C_27_H_36_O_14_ could correspond to sylvestrosides **III** and **IV** (**SIII** and **SIV**). These bis-iridoids contain a secoiridoid unit and an iridoid unit (Figure 7). Additionally, the second molecular formula was consistent with hemiacetal-secoiridoids (C_28_H_40_O_15_). This compound is likely an artefact formed during the treatment of **SIII** and/or **SIV** with methanol [50].

The ^1^H NMR spectrum of A2I displayed a signal of an aldehyde proton (δ_H_ 9.72), which is characteristic of these sylvestrosides (Figure S2e). The structural similarity between **SIII** and **SIV** gives rise to very complex ^1^H NMR spectra, making their differentiation difficult; however, comparison of the ^13^C NMR data with reported values allowed assignment, with the observed signals being consistent with **SIII** (Figure S2f) [30,33].

Meanwhile, the HRMS data of fraction A2II, obtained in both negative (Figure S3a) and positive (Figure S3b) ionization modes, together with its ^1^H NMR spectrum (Figure S3c), closely resembled those of fraction A2I. Furthermore, analysis of the ^13^C NMR spectrum (Figure S3d) indicated that fraction A2II was mainly composed of **SIV**. The signals (Table 2) were found to be consistent with values reported for this compound [30,33].

**Figure 8:**
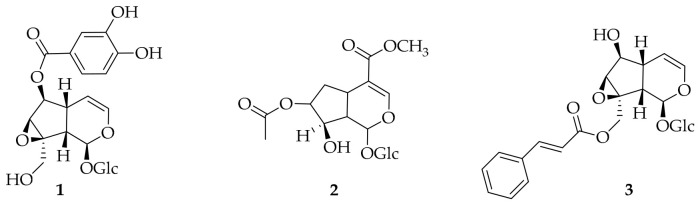
Glycoside iridoids with reported PPO inhibition properties.

Only a handful of iridoid glycosides have been evaluated as PPO/tyrosinase inhibitors to date, and the available evidence remains scarce. The reported activities vary markedly among structurally related compounds, indicating that PPO inhibition is not a general property of iridoid glycosides but rather depends on specific structural features. Among the few iridoid glycosides investigated, verproside (**1**, Figure 8) exhibited greater inhibitory activity than kojic acid against human tyrosinase, acting as a slow-binding competitive inhibitor through an enzyme isomerization mechanism [51]. Molecular docking and molecular dynamics simulations indicated stable binding within the enzyme active site through multiple hydrogen-bond interactions. Structure–activity analysis further suggested that the ortho-dihydroxy substitution pattern of the aromatic moiety plays a major role in inhibitory potency, while docking predicted interactions of the dihydroxyphenyl group with the catalytic copper ions. Another iridoid glycoside, 7-O-acetyl-4-O-acetate-1-β-D-glucopyranosyl iridoid (**2**, Figure 8), which lacks a dihydroxylated aromatic motif, showed competitive inhibition (Ki = 22.28 ± 0.73 μM), although docking analysis suggested that inhibition was not mediated by direct interactions with the catalytic copper ions [52]. Globularin (**3**, Figure 8) also displayed moderate tyrosinase inhibition (IC_50_ = 41.94 μM), whereas closely related iridoid glycosides were weakly active or inactive [53]. Likewise, newly isolated iridoid and bis-iridoid glycosides from *Scabiosa stellata* failed to inhibit tyrosinase despite their close structural similarity [54]. Collectively, these findings reinforce the conclusion that PPO inhibition within the iridoid family is highly structure-dependent and relies on specific molecular features that enable productive interactions with the enzyme active site.

A copper-chelation mechanism appears less likely to be a major contributor to PPO inhibition by sylvestrosides **III** and **IV**, as these compounds lack phenol or 3-hydroxy-4-keto motifs commonly associated with copper coordination in natural oxygenated tyrosinase inhibitors [55,56]. A notable structural difference between sylvestrosides **III** and **IV** and previously reported PPO-active iridoid glycosides is the presence of a terminal aldehyde functionality associated with the secoiridoid-derived moiety. Aldehydes have been exploited as electrophilic groups in the design of reversible covalent inhibitors, particularly for enzymes containing suitably positioned nucleophilic residues, where they can participate in reversible covalent interactions [57]. However, whether such chemical reactivity could contribute to PPO inhibition remains unknown. In the case of sylvestrosides **III** and **IV**, the aldehyde-bearing secoiridoid-derived moiety and the bis-iridoid architecture represent distinctive structural features that differentiate these compounds from previously reported PPO-active iridoid glycosides, thereby expanding the structural diversity of iridoid-type compounds associated with PPO inhibition.

It should be noted that although bioactivity-guided fractionation in the present study strongly associates PPO inhibitory activity with fractions enriched in sylvestrosides **III** and **IV**, the contribution of minor co-eluting metabolites cannot be completely excluded. Therefore, these results support the conclusion that sylvestrosides **III** and **IV** are likely major contributors to the observed PPO inhibition, while studies using individually purified compounds will be required to unequivocally establish their individual contributions and elucidate their mechanism of inhibition. The isolation of larger amounts of these compounds will also enable quantitative characterization and standardization of the active extract, supporting its future evaluation as a natural antibrowning agent.

### 2.4. Effect on Apple Slice Browning

To confirm the inhibitory effect on enzymatic browning observed in the bioautography assay, a preliminary experiment was conducted using fresh-cut apple slices. A *D. fullonum* leaf infusion extract (DF_L_-5) at 0.5% (*w*/*v*) was applied to Blue Bow Delicious apple slices, which were subsequently stored for eight days at 4 °C (Figure S4). The extract concentration was selected based on preliminary experiments, as higher concentrations produced a noticeable greenish coloration on the apple surface due to the natural pigments present in the extract. Water and 0.5% (*w*/*v*) ascorbic acid were used as negative and positive controls, respectively. Surface colour was measured by reflectance analysis and expressed in the CIELab colour space to calculate the browning index (BI).

**Figure 9:**
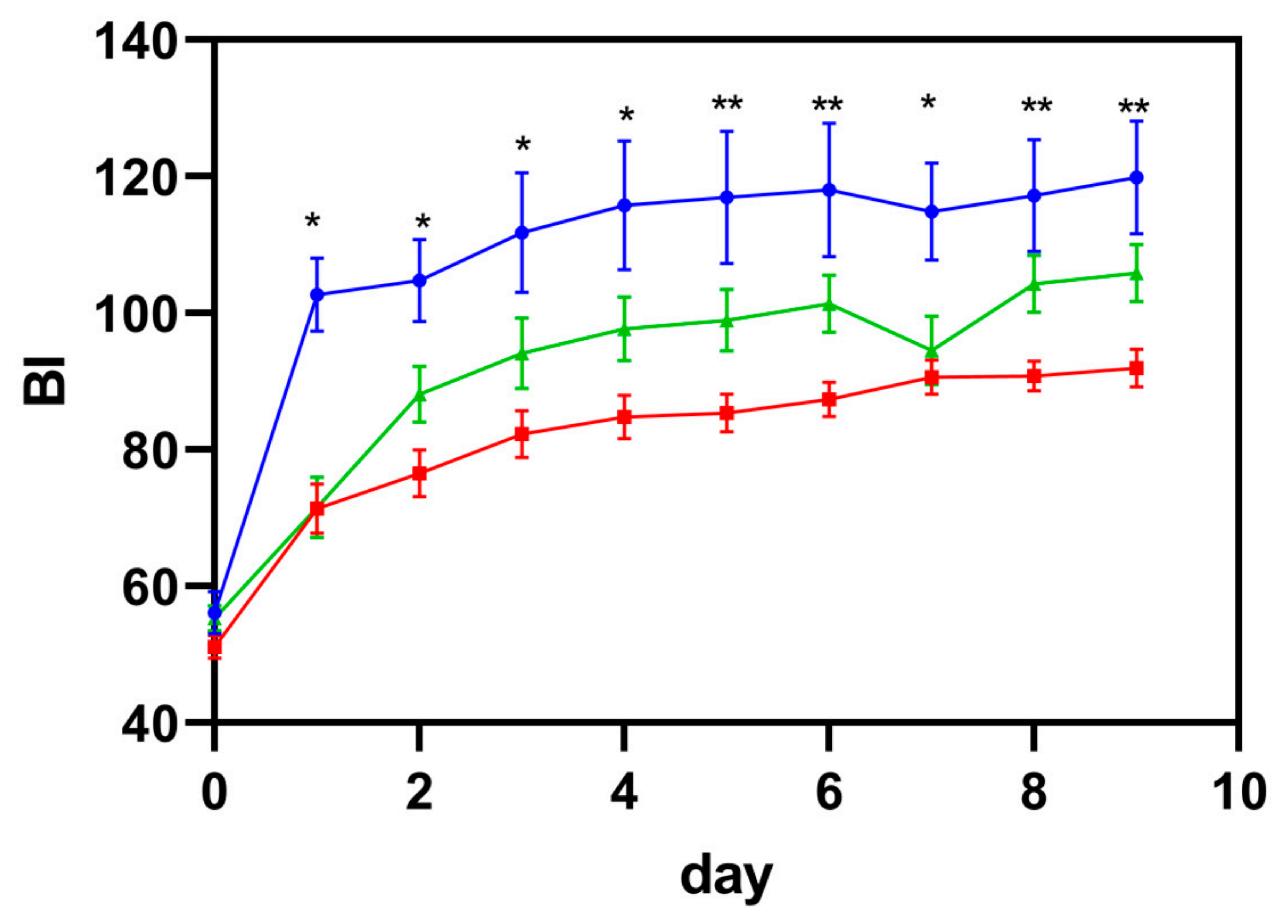
Evolution of BI during storage in apple slices. Treatments: water (blue circles), ascorbic acid (green triangles), and DF-5 extract (red squares). Error bars represent the standard error of the mean. Results were analyzed using a mixed-effect model, and differences between treatments were assessed at each time point. Asterisks indicate significant differences between treatments on the same day (*p* > 0.05). * Significant difference between DF-5 and water. ** Significant differences between DF and both water and ascorbic acid.

Figure 9 shows the evolution of BI along storage time. A sharp increase in BI was observed on the first day in water-treated samples, whereas both the DF-5 and ascorbic acid delayed this increase. Apple slices treated with DF_L_-5 consistently exhibited lower BI values than water-treated samples throughout the eight-day storage period. From day five onwards, BI values for DF-treated slices were significantly lower than those observed for ascorbic acid-treated samples (indicated with **), except on day seven. These results indicate that DF-5 extract effectively reduces browning in fresh-cut apple slices stored at 4 °C in sealed bags, which represents a common storage and commercialization condition for this type of product.

While the bioautography-guided approach identified PPO inhibition as a major mechanism underlying this effect, the overall antibrowning activity observed in fresh-cut apples is probably the result of complementary mechanisms. In addition to PPO inhibition, other constituents present in the crude infusion extract may contribute to browning reduction through antioxidant mechanisms, such as scavenging reactive oxygen species or reducing quinone intermediates formed during enzymatic oxidation. Furthermore, while the bioactivity-guided fractionation strongly associates PPO inhibitory activity with fractions enriched in sylvestrosides **III** and **IV**, the possible contribution of minor co-eluting metabolites cannot be excluded. Therefore, the antibrowning effect observed in the food matrix may arise from the combined action of PPO inhibition and antioxidant mechanisms.

Although microbiological analyses were not performed, no visible signs of microbial contamination were observed on the surface of the apple slices after eight days of storage. This finding is consistent with previous reports describing the antimicrobial activity of *D. fullonum* leaf extracts [31]; however, further microbiological studies would be required to confirm this effect.

These results demonstrate the potential of *D. fullonum* extracts as natural antibrowning agents. Nevertheless, several aspects should be considered before industrial application. Plant extracts may influence sensory attributes, including flavour, aroma, or colour, depending on their composition and the food matrix. Moreover, the chemical composition of botanical extracts can vary according to plant origin, harvest conditions, and extraction procedure, highlighting the need for standardized production. From a regulatory perspective, additional toxicological and safety assessments would also be required before use as food additives or processing aids. Taken together, these findings support the potential of *D. fullonum* extracts as natural antibrowning agents. However, further studies addressing sensory properties, extract standardization, safety, and process scalability will be necessary before practical food applications can be considered.

## 3. Materials and Methods

### 3.1. Chemicals

3-Methyl-2-benzothiazolinone hydrazone (MBTH), 2,2-diphenyl-1-picrylhydrazyl (DPPH), poly(vinylpolypyrrolidone) (PVPP), 2,2′-azino-bis(3-ethylbenzothiazoline-6-sulfonic acid (ABTS), 6-hydroxy-2,5,7,8-tetramethylchroman-2-carboxylic acid (Trolox), 2,9-dimethyl-1,10-phenanthroline (neocuproine), copper (II) chloride, Viscozyme L, and catechol were purchased from Sigma (St Louis, MO, USA). 3,4-dihydroxy-L-phenylalanine (L-DOPA) was purchased from Saporiti (Buenos Aires, Argentina). Folin–Ciocalteu reagent, gallic acid, ethanol, acetone, chloroform, ethyl acetate, ascorbic acid (AA), calcium carbonate, disodium hydrogen phosphate, and sodium dihydrogen phosphate were purchased from Cicarelli (Rosario, Argentina). Methanol, Ammonium acetate, and potassium persulfate were purchased from Biopack (Buenos Aires, Argentina). Aluminum-backed silica gel 60 F254 TLC layers were purchased from Merck (Darmstadt, Germany). Silica was acquired from Analtech (Newark, NJ, USA). Agarose was purchased from Biodynamics (Buenos Aires, Argentina). All reagents used were analytical grade.

### 3.2. Plant Material

Aerial parts of wild-growing specimens of the species were collected at Pergamino, Buenos Aires, Argentina. A voucher specimen for each plant species was stored at the herbarium from Universidad Nacional de Rosario, Argentine: *A. sativum* (Amaryllidaceae), ID: commercial (AS); *B. rapa* (Brassicaceae), ID: MO003 (BR); *C. bursa-pastoris* (Brassicaceae), ID: MO043 (CBP); *C. bonariensis* (Brassicaceae), ID: MO041 (CB); *C. rivulariastrum* (Caryophyllaceae), ID: MO044 (CR); *C. intybus* (Asteraceae), ID: MO051 (CI); *D. fullonum* (Dipsacaceae), ID: MO045 (DF); *E. plantagineum* (Boraginaceae), ID: MO032 (EP); *E. malacoides* (Geraniaceae), ID: MO042 (EM); *E. horridum* (Apiaceae), ID: MO058 (EH); *L. amplexicaule* (Lamiaceae), ID: MO001 (LA); *L. multiflorum* (Poaceae), ID: MO031 (LM); *M. recutita* L. (Asteraceae), ID: MO008 (MR); *M. lupulina* (Fabaceae), ID: MO037 (ML); *N. longiflora* (Solanaceae), ID: MO046 (NL); *O. articulata* (Oxalidaceae), ID: MO030 (OA); *P. caerulea* (Passifloraceae), ID: MO057 (PC); *R. sativus* (Brassicaceae), ID: MO049 (RS); *R. rugosum* (Brassicaceae), ID: MO014 (RR); *S. rhombifolia* (Malvaceae), ID: MO050 (SR); *S. arvensis* (Brassicaceae), ID: MO022 (SA); *S. sisymbriifolium* (Solanaceae), ID: MO011 (SS); *S. oleraceus* (Asteraceae), ID: MO013 (SO); *S. bonariensis* (Malvaceae), ID: MO018 (SB); *U. urens* (Urticaceae), ID: MO010 (UU); *Z. mays* (estigma) (Poaceae), ID: commercial (ZM).

The collected plant material was dried at 40 °C for three days in a forced air current oven and then ground in a Willey cutting mill equipped with a 2 mm screen (Thomas Scientific, Swedesboro, NJ, USA).

Blue Bow Delicious apples were purchased from the local market.

### 3.3. Extract Preparation

#### 3.3.1. General Screening Protocols

Extracts for initial screening were prepared from the aerial parts of the different plant species.

**Protocols 1 and 2:** Plant material was extracted under reflux with 96% ethanol (Protocol 1) or methanol (Protocol 2) for 45 min, and the extraction procedure was repeated three times. The first extraction was performed using 5 mL of solvent per gram of plant material. For the two subsequent extractions, a solvent volume tenfold lower than that used in the first extraction. Plant residues were removed by filtration, and the crude ethanolic or methanolic extracts were pooled and concentrated under reduced pressure to constant weight.

**Protocol 3:** A total of 1.50 g of plant material was hydrated with 13.00 mL of 0.10 M citrate/citric acid buffer (pH 4.5). Subsequently, an additional 0.10 M citrate/citric acid buffer (pH 4.5) was added to obtain a final concentration of 5.0% (*w*/*v*). The mixture was maintained at 45 °C for 90 min.

**Protocol 4.** A total of 1.50 g of plant material was hydrated with 13 mL of 0.10 M citrate/citric acid buffer (pH 4.5). Then, fungal Viscozyme (Novozymes, Bagsværd, Denmark) was added to achieve a final enzyme activity of 6.0 Fungal Beta-Glucanase units/g dry material. The resulting mixture was ground for 5 min in a mortar. Subsequently, an additional 0.10 M citrate/citric acid buffer (pH 4.5) was added to obtain a final extract concentration of 5.0% (*w*/*v*). The mixture was maintained at 45 °C for 90 min. At the end of the treatment, carbohydrolase enzymes were inactivated by heating at 90 °C for 1 min.

**Protocol 5:** 4.5 g of dried plant material was added to 90 mL of boiling water, and the mixture was allowed to stand covered under constant stirring for 10 min. The extractive solution was then filtered and lyophilized to obtain a dry extract.

**Protocol 6:** 4.50 g of dried plant material was added to 90 mL of water, and the mixture was kept boiling under constant stirring for 10 min. The extractive solution was then filtered and lyophilized to obtain a dry extract.

**Protocol 7:** 4.50 g of dried plant material was added to 90 mL of water, and the mixture was kept at room temperature under constant stirring for 3 days. The extractive solution was then filtered and lyophilized to obtain a dry extract.

#### 3.3.2. Preparation of DF_L_-5, DF_L_-6, DF_s_-6, DF_F_-6 and DF_L_-8 Extracts

**Infusion protocol for DF_L_-5 preparation:** 1.00 g of dried leaves was added to 20.00 mL of boiling water, and the mixture was allowed to stand covered for 10 min. The extractive solution was then filtered and lyophilized to obtain 0.30 g of dry extract (30.0% yield).

**Decoction protocol for DF_L_-6, DF_s_-6, DF_F_-6 preparation:** 1.00 g of dried plant part (leaves, stems or flower receptacles) was added to 20.00 mL of water, and the mixture was kept boiling for 10 min. The extractive solution was then filtered and lyophilized to obtain 0.26 g of dry extract (DF_L_-6: 26.0% yield, DF_S_-6: 9.33%, DF_F_-6: 6.66%).

**Ultrasound-assisted ethanolic extraction protocol for DF_L_-8 preparation**: The method described by Saar-Reismaa et al. [31] was followed with slight modifications. Briefly, 1.00 g of dried leaves was mixed with 5.00 mL of 70% ethanol and shaken for 25 min. The mixture was then sonicated at 40 °C for 30 min and filtered. The residue was subjected to a second extraction under the same conditions. Both extractive solutions were combined, and solvents were removed first under reduced pressure using a rotary evaporator (Senco Technology Co., Sanghái, China) and subsequently by lyophilization, yielding 0.10 g of dry extract (10.0% yield).

#### 3.3.3. Scale-Up of DF_L_-5 Extract

To obtain sufficient material for compound purification, a larger-scale infusion extraction was performed by adding 4.00 L of boiling water to 200.00 g of dried leaves. The mixture was allowed to stand for 10 min and then filtered twice. The resulting extract solutions were lyophilized to constant weight, yielding 79.90 g of dry extract (39.9% yield).

### 3.4. Chromatography and Image Analysis of Extracts

TLC separation of natural extracts was performed on 7 × 5 cm aluminum-backed silica gel 60 F254 plates. A 2.5 μL aliquot of each sample (400 μg) was applied as 4 mm bands on the TLC plate. Elution was carried out with chloroform:methanol:water (85:25:1). The TLCs were performed with a HPTLC system (CAMAG, Muttenz, Switzerland) consisting of an automatic TLC Sampler 4 (ATS 4), a TLC visualizer, and vision CATS v3.2.23051.1 software. All plates were documented using a TLC Visualizer (CAMAG, Muttenz, Switzerland) under visible light, UV light at 366 nm or 254 nm, as appropriate.

For the detection of iridoids, the plates were sprayed with vanillin–sulphuric acid reagent.

### 3.5. Autographic Assays

Study of enzymatic browning inhibition activity and antioxidant activity was carried out by TLC coupled autographic assays.

#### 3.5.1. TLC-PPO Assay

**Preparation and activity quantification of apple PPO**: PPO was extracted from Blue Bow Delicious apples purchased on the local market. The extraction was performed by homogenizing 50 g of peeled apple cuts with a home mixer in 50 mL of 50 mM sodium phosphate buffer (pH 6.8). 4% *w*/*v* PVPP was added to adsorb endogenous polyphenols. The procedure was carried out at 4 °C. The extract was clarified by centrifugation, and the supernatant was stored at −20 °C for further experiments. Enzyme activity was quantified after extraction by measuring the increase in absorbance at 420 nm using catechol as substrate in phosphate buffer at pH 6.8 at 25 °C [28]. One enzymatic unit (U) was defined as an increase in absorbance of 0.001/min. Assays were run in triplicate, and results averaged.

**Analysis of extracts by TLC-PPO assay:** An autographic assay for the detection of apple PPO inhibitors was conducted on TLC plates following the method described by Micheloni et al. [28]. The assay was performed as follows, with all quantities expressed as mass of reagent or volume per cm^2^ of TLC plate. Agar (1.05 mg) and MBTH (10 μg) were dissolved in 0.13 mL of 50 mmol/L phosphate buffer (pH 6.8) at approximately 80 °C. Subsequently, 20 μL of a 15 mmol/L aqueous L-DOPA solution was added. The agar solution was allowed to cool to 45 °C, after which 2.71 μL of PPO solution (4.0 U) was added, and the mixture was thoroughly homogenized. The resulting agar-containing revealing medium was applied onto the TLC plate and allowed to solidify at ambient temperature, forming the immobilized enzymatic system. In this system, PPO catalyzes the oxidation of L-DOPA to dopaquinone, which reacts with MBTH to form a reddish-coloured complex. PPO inhibition zones are visualized as white spots against the reddish background of the plate. To confirm that the observed inhibition zones were associated with PPO inhibition rather than nonspecific reactions, a control experiment was performed in the absence of the enzyme. For this purpose, the agar-containing revealing medium was prepared following the same procedure, except that PPO was omitted. To conduct a rapid screening of apple PPO inhibition, the test was performed without prior chromatographic development. 400 μg of the whole plant extract from each collected species was loaded as spots onto a TLC plate. The positive DF extracts were further studied by developing TLC plates. Extracts were applied in bands, and TLCs were developed as described in Section 2.3. After that, the solvent was evaporated, a revealing system was poured on the TLC plate, and an apple PPO inhibition test was carried out on the developed plate. Assays were run in duplicate.

**Dose–response analysis of the DF-6 extract:** TLC plates were spotted with increasing amounts of the DF-6 extract (100–400 µg) and subsequently evaluated using the PPO–TLC assay. Plates were imaged using a TLC Visualizer (CAMAG, Muttenz, Switzerland), and the resulting images were imported into ImageJ 1.53c (Fiji) for semi-quantitative analysis of the inhibition halos. Images were split into individual colour channels, and the blue channel was selected for its higher contrast between the halo and the background. The blue channel was converted to 8-bit and binarized using the Default thresholding algorithm; internal gaps corresponding to the inoculation marker were removed using the Fill Holes function. For each binarized halo, the diameter was measured at twelve different angles (15° intervals, 0–165°) through the spot centroid, and the mean diameter and standard deviation (SD) were calculated for each spot. The dose–response relationship was assessed by plotting the mean halo diameter (±SD) against the amount of extract applied to the plate (Figure 2) and Table S1 in Supplementary Material.

#### 3.5.2. Determination of Antioxidant Capacity

TLC assays for the detection of DPPH, ABTS^•+^ radical scavenging activity, and CUPRAC reducing capacity were performed as reported by de Torre et al. [58], Zampini et al. [49], and Micheloni et al. [59], respectively. TLC plates were run in duplicate.

### 3.6. Spectrophotometric Measurements

#### 3.6.1. PPO Inhibitory Activity Assay

The inhibitory effect on apple PPO activity was quantified by measuring the residual enzymatic activity (REA %) [60]. Apple PPO was obtained as described in Section 3.5.1. The enzymatic reaction was initiated by adding 1 mL of 0.2 M catechol solution to 2 mL of enzyme solution consisting of 100 µL of PPO solution, 100 µL of test sample, and 1.8 mL of 50 mM phosphate buffer (pH 6.8). Prior to the reaction, the enzyme solution was incubated with different extract concentrations (0.8–4.5 mg/mL) for 8 min at 25 °C. Absorbance at 420 nm was continuously recorded for 2 min at 25 °C using a Lambda 25 spectrophotometer (PerkinElmer, Shelton, CT, USA). Only the linear portion of the absorbance versus time curve was considered for enzyme activity calculations.

Residual enzymatic activity was calculated according to the following equation: REA = ∆As/∆Ao × 100, where ∆Ao represents absorbance change at 420 nm without test sample, and ∆As represents the absorbance change in the presence of the test sample. REA (%) values were plotted against the logarithm of test sample concentration, and the concentration required to reduce REA to 50% (IC_50_) was determined.

#### 3.6.2. Total Phenolic Content

The total phenolic content was determined using Folin–Ciocalteu reagent and gallic acid as standard [61]. A 2.4 mL aliquot of sample solution (0.083 mg/mL) was mixed with 0.2 mL of Folin–Ciocalteu phenol reagent. After 5 min, 10% *w*/*v* calcium carbonate (2.4 mL) was added, and the mixture was kept in total darkness for 20 min at ambient temperature. The absorbance was measured at 760 nm using a Lambda 25 spectrophotometer (Perkin Elmer, Springfield, IL, USA). Phenolic acid content was calculated using a gallic acid calibration curve from 0.1 to 0.4 mg/mL and the results expressed as gallic acid equivalents in mg/g of dry matter.

#### 3.6.3. ABTS^•+^ Radical Scavenging Assay

Free radical scavenging capacity was evaluated by measuring the bleaching of a blue-coloured ABTS^•+^ solution according to the method described by Zampini et al. [49]. ABTS^•+^ was generated by reaction with sodium persulfate to obtain final concentrations of 7 mM ABTS and 2.45 mM sodium persulfate. The mixture was stirred continuously in the dark for 16 h and then diluted with water to an absorbance of 0.7.

Subsequently, 0.015 mL of sample solutions (0.026–0.35 mg/mL) were added to 0.6 mL of ABTS^•+^ solution. The reaction mixture was incubated in the dark for 30 min, and absorbance was measured at 756 nm. The percentage of radical scavenging activity was calculated as: ((Ao − Ae)/Ao) × 100, where Ao represents the absorbance in the absence of sample and Ae represents the absorbance in the presence of sample. The IC_50_ value (mg/mL) was then determined.

#### 3.6.4. DPPH Radical Scavenging Assay

The free radical scavenging capacity of the extracts was evaluated based on the bleaching of a purple methanolic solution of the DPPH radical. The method described by Kubola & Siriamornpun [62] was used with slight modifications. Briefly, 0.015 mL of sample solutions at different concentrations (0.026–0.35 mg/mL) were added to 1 mL of 0.0003% DPPH solution. A blank sample without extract was also prepared. Absorbance at 517 nm was measured after 30 min of incubation at room temperature in the dark, and the percentage of radical scavenging activity was calculated as ((Ao − Ae)/Ao) × 100, where Ao represents the absorbance in the absence of sample and Ae represents the absorbance in the presence of sample. The concentration required to scavenge 50% of the DPPH radical (IC_50_) was then determined.

#### 3.6.5. CUPRAC Assay

To quantify the Cu(II)-reducing capacity of the extracts previously detected by TLC autography, the method described by Özyürek et al. [63] was used. Briefly, the reaction mixture was prepared by mixing 0.06 mL of sample solution (200 mg/mL) with 0.11 mL of H_2_O, followed by the addition of 0.17 mL each of Cu(II) aqueous solution (0.02 M), ammonium acetate buffer (1 M, pH 7), and neocuproine ethanolic solution (7.5 mM), to obtain a final volume of 0.68 mL. The tubes were stoppered and, after 30 min, the absorbance at 450 nm was measured using a Lambda 25 spectrophotometer against a reagent blank. Trolox^®^ equivalent content was determined using an external calibration curve prepared with Trolox^®^ concentrations ranging from 0.0027 to 0.16 mg/mL, and the results were expressed as mg Trolox^®^ equivalents per g of dry matter.

All the assays based on spectrophotometric measurements were performed in duplicate, and results were averaged.

### 3.7. Purification of DF Extract

A total of 10.00 g of the extract was subjected to flash silica gel column chromatography using chloroform:methanol mixtures of increasing polarity as eluents (from 90:10 to 50:50), yielding ten fractions (368 mL each). Solvents were removed under reduced pressure, and the fractions were evaluated by TLC-PPO assay (0.40 mg applied per spot) on silica gel plates using chloroform:methanol (85:15) as the mobile phase. Fractions 3 and 4, which showed the highest PPO inhibitory activity, were combined and designated as fraction A (0.34 g, 3.4%).

Fraction A was further subjected to silica gel column chromatography using chloroform:methanol mixtures of increasing polarity (from a 90:10 to 85:15). A total of 97 fractions (5 mL each) were collected. Apple PPO inhibitory activity was detected in fractions 14 to 29. Fractions 15 to 19 (15 mg) were pooled (A2I), and preparative TLC was performed three times using dichloromethane:methanol (89:11) to obtain three fractions. The less polar fraction was enriched with sylvestroside IV (5.00 mg, 0.05%). Similarly, fractions 20 to 25 (11 mg) were pooled (A2II), and preparative TLC was performed four times using dichloromethane:methanol (89:11) to obtain two fractions. The most polar fraction was enriched with sylvestroside **III** (4.00 mg, 0.04%).

### 3.8. Mass Spectrometry and Nuclear Magnetic Resonance

Proton nuclear magnetic resonance (^1^H NMR) spectra were recorded on a Bruker Advance II (Bruker, Billerica, MA, USA) at 300 MHz in (CD_3_)_2_CO using tetramethylsilane (TMS) (0.00 ppm) as the internal standard. ^13^C NMR spectra were recorded on the same instrument at 75 MHz with (CD_3_)_2_CO with TMS (0.00 ppm) as the internal standard.

High-resolution mass spectra (direct infusion MS experiments) were recorded on a Q-Exactive (Thermo Fisher Scientific, Waltham, MA, USA). Methanol was used for sample preparation. MS: source type, electrospray ionization (ESI); positive ion mode; nebulizer pressure, 0.4 Bar; gas dry temperature, 180 °C; dry gas flow, 4.0 L/min; capillary voltage, 4500 V; end plate offset, 500 V; collision cell radio frequency, 150.0 Vpp. ISC.

### 3.9. Superficial Colour Measurement

Fresh-cut apples were immersed for 8 min in a 0.5% (*w*/*v*) aqueous solution of the dry DF_L_-5 extract obtained by infusion. After treatment, the apple slices were drained on absorbent paper and placed in resealable polyethylene bags. Fresh-cut apples immersed in distilled water and in a 0.5% (*w*/*v*) aqueous solution of ascorbic acid were used as negative and positive controls, respectively. All samples were stored at 4 °C for 8 days, and surface colour was monitored during storage. Surface colour was measured in CIELab colour space by using a Miniscan EZ colorimeter (Hunter Lab, Reston, VA, USA) configured at 2° observer angle and D65 standard light source. For each treatment, fourteen slices were analyzed as experimental replicates. For each slice, three analytical measurements were taken by rotating the colorimeter 120° to overcome surface irregularities, and the results were averaged. From surface colour data, the browning index (BI) was calculated as follows [64].

BI = [100 (x − 0.31)]/0.172

where x = (a* + 1.75L*)/(5.645L* + a* − 3.012b*) and L*, a* and b* are colour coordinates in CIELab space.

### 3.10. Statistical Analysis

ANOVA with Tukey post hoc test was conducted to establish statistical differences. InfoStat version 212 (Grupo InfoStat, FCA, Universidad Nacional de Córdoba, Argentina) was used for statistical analysis. GraphPad Prism version 6.00 for Windows (San Diego, CA, USA) was used for graphical analysis on the superficial colour measurement experiment.

## 4. Conclusions

This study demonstrates the usefulness of a TLC-based bioautographic assay using apple-derived polyphenol oxidase (PPO) for the targeted discovery of natural antibrowning agents from plant sources. Among the species evaluated, *D. fullonum* leaf infusions exhibited the strongest PPO inhibitory activity, highlighting the importance of both plant organ and extraction method in determining bioactivity. The results further indicate that PPO inhibition is associated with specific metabolites rather than with a general antioxidant effect.

Bioactivity-guided fractionation associated the inhibitory activity with fractions enriched in the iridoid glycosides sylvestrosides **III** and **IV**, suggesting that these metabolites are likely major contributors to the observed PPO inhibition. Although further studies using individually purified compounds are required to confirm their individual contributions and elucidate their mechanism of action, the present findings demonstrate the effectiveness of target-oriented bioautographic screening for the identification of bioactive constituents from complex plant extracts.

Importantly, the PPO inhibitory activity observed in vitro translated into effective browning control in fresh-cut apples during refrigerated storage, where the *D. fullonum* leaf infusion showed antibrowning performance comparable to that of ascorbic acid. Overall, this work identifies *D. fullonum* leaves as a promising source of natural antibrowning agents and provides a practical workflow for the rapid discovery of PPO inhibitors with potential applications in minimally processed foods.

## Figures and Tables

**Table 1:** Radical scavenging capacity for DPPH or ABTS^•+^ assays, expressed as the concentration of extract (mg/mL) required to scavenge 50% of radicals (IC_50_), CUPRAC (Cu(II) reduction capacity) expressed as milligrams of Trolox^®^ equivalents per gram of dry material (mgTL/gDM), and Total Phenols expressed as milligrams of gallic acid equivalents per gram of dry plant material (mgGA/gDM). Values are expressed as mean ± SD.

Sample	DPPH (mg/mL)	ABTS^•+^ (mg/mL)	CUPRAC (mgTL/gDM)	Total Phenols (mgGA/gDM)
DF-6	0.12 ± 0.01 ^a^	0.15± 0.01 ^a^	0.530 ± 0.001 ^a^	11.09 ± 0.06 ^b^
DF_L_-6	0.10 ± 0.01 ^a^	0.081 ± 0.004 ^b^	0.277 ± 0.036 ^b^	18.66 ± 0.03 ^d^
DF_F_-6	0.12 ± 0.01 ^a^	0.090 ± 0.005 ^c^	0.328 ± 0.018 ^b^	12.82 ± 0.06 ^c^
DF_s_-6	0.53 ± 0.04 ^b^	0.31 ±0.02 ^d^	0.145 ± 0.001 ^c^	6.71 ± 0.04 ^a^
ascorbic acid	0.016 ± 0.001	0.01 ± 0.01	—	—
Trolox^®^	0.0063 ± 0.0003	0.0024 ± 0.0002	—	—

Different letters (a–d) indicate statistically significant differences among extracts (*p* < 0.05) after ANOVA followed by Tukey post-oc test. Ascorbic acid and Trolox^®^ were included as standards to compare scavenging activity but excluded from the statistical analysis to allow comparison among plant extracts.

**Table 2:** ^13^C NMR chemical shifts in the active fractions A2_I_ and A2_II_.

Fraction	Unit	Carbon											
		C1	C2	C3	C4	C5	C6	C7	C8	C9	C10	C11	C12
A2_I_	a	96.8		152.8	110.0	27.1	44.7	201.3	134.8	44.8	120.2	166.8	
	b	96.6		153.0	111.5	33.3	40.4	77.2	41.3	48.1	14.3	168.1	51.3
	Glu	99.8	74.5	77.9	71.5	78.1	62.9						
A2_II_	a	96.8		153.3	109.8	27.2	44.8	201.2	134.9	45.0	120.3	166.8	
	b	70.0		169.8	52.2	37.4	38.9	79.3	41.9	42.8	13.5	169.5	52.8
	Glu	99.8	74.5	77.9	71.5	78.1	62.9						

Solvent: (CD_3_)_2_CO (75 MHz).

## Data Availability

The original contributions presented in this study are included in the article/supplementary material. Further inquiries can be directed to the corresponding authors.

## Supplementary Materials

The following supporting information can be downloaded at: https://www.mdpi.com/article/10.3390/plants15152374/s1, Figure S1: TLC chromatograms of DF extracts (400 µg): aqueous decoction extract (DF-6, track 1), leaves extract (DFL-6, track 2), flower receptacles extract (DFF-6, track 3), and stems extract (DFS-6, track 4). Observed with DPPH (a), ABTS•+ (b), and CUPRAC-TLC assays (c). The mobile phase used was chloroform:methanol:water (85:25:1); Figure S2: Fraction A2I: (a) mass spectrum in negative mode, (b) zoom mass spectrum in negative mode for m/z 583.2046, (c) zoom mass spectrum in negative mode for m/z 615.2308, (d) mass spectrum in positive mode, (e) 1H NMR spectrum (300 MHz) and, (f) 13C NMR spectrum (75 MHz); Figure S3: Fraction A2II: (a) negative-ion mass spectrum, (b) positive-ion mass spectrum, (c) 1H NMR spectrum (300 MHz) and, (d) 13C NMR spectrum (75 MHz); Figure S4: Representative images of apple slices treated with DF infusion extract (DF-5), ascorbic acid, or water during 9 days of storage in sealed polyethylene bags at 4 °C; Table S1: Dose–response analysis of the DF-6 extract by the PPO–TLC assay. Halo diameters were measured on ImageJ 1.53c-binarized images at twelve angles (15° intervals) through the spot centroid; values represent the mean ± standard deviation (SD) of these twelve diametral measurements per spot.

## Abbreviations

The following abbreviations are used in this manuscript:

A2I	Fraction A2I
A2II	Fraction A2II
AA	Ascorbic acid
ABTS	2,2′-azino-bis(3-ethylbenzothiazoline-6-sulfonic acid
ABTS^+^	2,2′-azino-bis(3-ethylbenzothiazoline-6-sulfonic acid radical proton
Ae	Absorbance in the presence of sample
ANOVA	Statistical analysis of variance
Ao	Absorbance in the absence of sample
AS	*Allium sativum* L.
BI	Browning index
BR	*Brassica rapa* L.
CB	*Cardamine bonariensis* Pers. var.* bonariensis*
CBP	*Capsella bursa-pastoris* (L.) Medik.
CI	*Cichorium intybus* L.
CIELab	CIELab colour space
CO	Catechol oxidase
CR	*Cerastium rivulariastrum* Moeschi & Pedersen
CUPRAC	Cupric Reducing Antioxidant Capacity
DF	*Dipsacus fullonum* L.
DF_F_	Extract of *Dipsacus fullonum* L. flower receptacles
DF_L_	Extract of *Dipsacus fullonum* L. leaves
DF_S_	Extract of *Dipsacus fullonum* L. stems
DPPH	2,2-diphenyl-1-picrylhydrazyl
EH	*Eryngium horridum* Malme
EM	*Erodium malacoides* (L.) L’Hér.
EP	*Echium plantagineum* L.
ESI	Electrospray ionization
gDM	Gram of dry material
Glu	Glucose
HPTLC	High-Performance Thin-Layer Chromatography
HRMS	High-Resolution Mass Spectrometry
IC_50_	Half Maximal Inhibitory Concentration
L-DOPA	3,4-dihydroxy-L-phenylalanine
LA	*Lamium amplexicaule* L.
LM	*Lolium multiflorum* Lam.
M	Molar concentration, mol/L
MBTH	3-Methyl-2-benzothiazolinone hydrazone
mDA	Millidalton
mgTL	Milligrams of Trolox^®^ equivalents
mgGA	Milligrams of gallic acid equivalents
ML	*Medicago lupulina* L.
mM	Millimolar concentration, mmol/L
MR	Matricaria recutita L.
MS	Mass Spectrometry
NL	*Nicotiana longiflora* Cav.
OA	*Oxalis articulata* Savigny
PC	*Passiflora caerulea* L.
PPO	Polyphenol oxidase
PVPP	Poly(vinylpolypyrrolidone)
REA	Residual enzymatic activity
*Rf*	Retardation factor
RR	*Rapistrum rugosum* L.
RS	*Raphanus sativus* L.
**SIII**	Sylvestroside **III**
**SIV**	Sylvestroside **IV**
SA	*Sinapis arvensis* L.
SB	*Sphaeralcea bonariensis* (Cav.) Griseb.
SD	Standard deviation
SO	*Sonchus oleraceus* L.
SR	*Sida rhombifolia* L.
SS	*Solanum sisymbriifolium* Lam. var. *sisymbriifolium*
TLC	Thin-layer chromatography
TL	Trolox^®^
TROLOX	6-hydroxy-2,5,7,8-tetramethylchroman
UV	Ultra violet
TMS	Tetramethylsilane
UU	*Urtica urens* L.
ZM	*Zea mays* L.
