# Supplementary material for: Characterization of Dipsacus fullonum L. Extract and Its Active Components as Natural Additives to Prevent Enzymatic Browning
Source: Plants (Basel). 2026 Aug 3;15(15):2374. doi: 10.3390/plants15152374 (PMC13468179; doi:10.3390/plants15152374)
Supplement: Supplementary file 1 [file plants-15-02374-s001.zip › plants-4420646-supplementary.pdf]

Supplementary Information for

Characterization of *Dipsacus fullonum* L. extract and its active components as natural additives to prevent enzymatic browning

Oscar B. Micheloni [1,4], Mario O. Salazar [2,4], I. Ayelen Ramallo [2], Abel E. Farroni [3,,*] and Ricardo L. E. Furlan [2,*]

[1] Departamento de Ciencias Básicas, Universidad Nacional del Noroeste de la Provincia de Buenos Aires, Monteagudo 2772, 2700, Pergamino, Buenos Aires, Argentina; obmicheloni@comunidad.unnoba.edu.ar
[2] Área Farmacognosia, Facultad de Ciencias Bioquímicas y Farmacéuticas. Universidad Nacional de Rosario-CONICET, Suipacha 531, S2002LRK, Rosario, Santa Fe, Argentina; msalazar@fbioyf.unr.edu.ar, aramallo@fbioyf.unr.edu.ar, and rfurlan@fbioyf.unr.edu.ar
[3] Laboratorio de Biotecnología, EEA Pergamino, Instituto Nacional de Tecnología Agropecuaria. Av. Frondizi Km 4.5 Pergamino, Buenos Aires, Argentina, CC 31 - B2700WAA; farroni.abel@inta.gob.ar
[4] Both authors contributed equally
* Correspondence: farroni.abel@inta.gob.ar, rfurlan@fbioyf.unr.edu.ar, furlan@rosario-conicet.gov.ar

Contents

Figure S1: TLC chromatograms of DF extract … S
Figure S2: Fraction A2I … S
Figure S3: Fraction A2II … S
Figure S4: Effect on apple slice browning… S
Table S1: Dose–response analysis of the DF-6 extract by the PPO–TLC assay… S

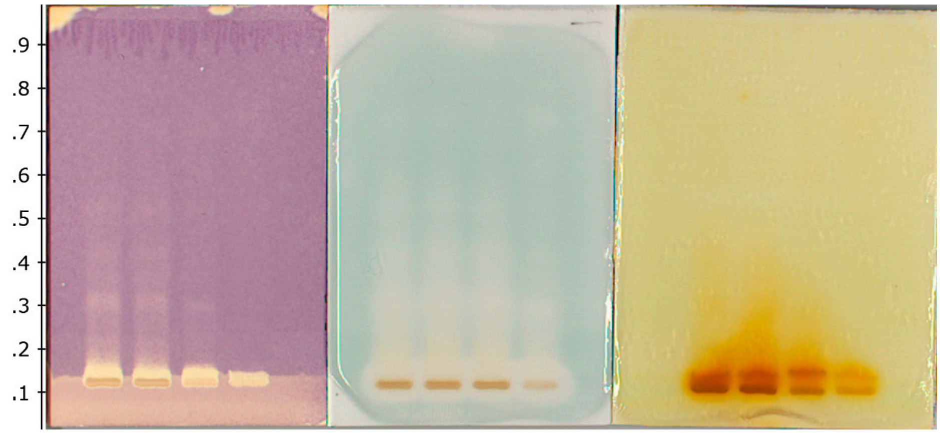

Figure S1: TLC chromatograms of DF extracts (400 μg): aqueous decoction extract (DF-6, track 1), leaves extract (DF_L-6, track 2), flower receptacles extract (DF_F-6 track 3), and stems extract (DF_S-6, track 4). Observed with DPPH (a), $ABTS^{•+}$ (b), and CUPRAC-TLC assays (c). The mobile phase used was chloroform:methanol:water (85:25:1).

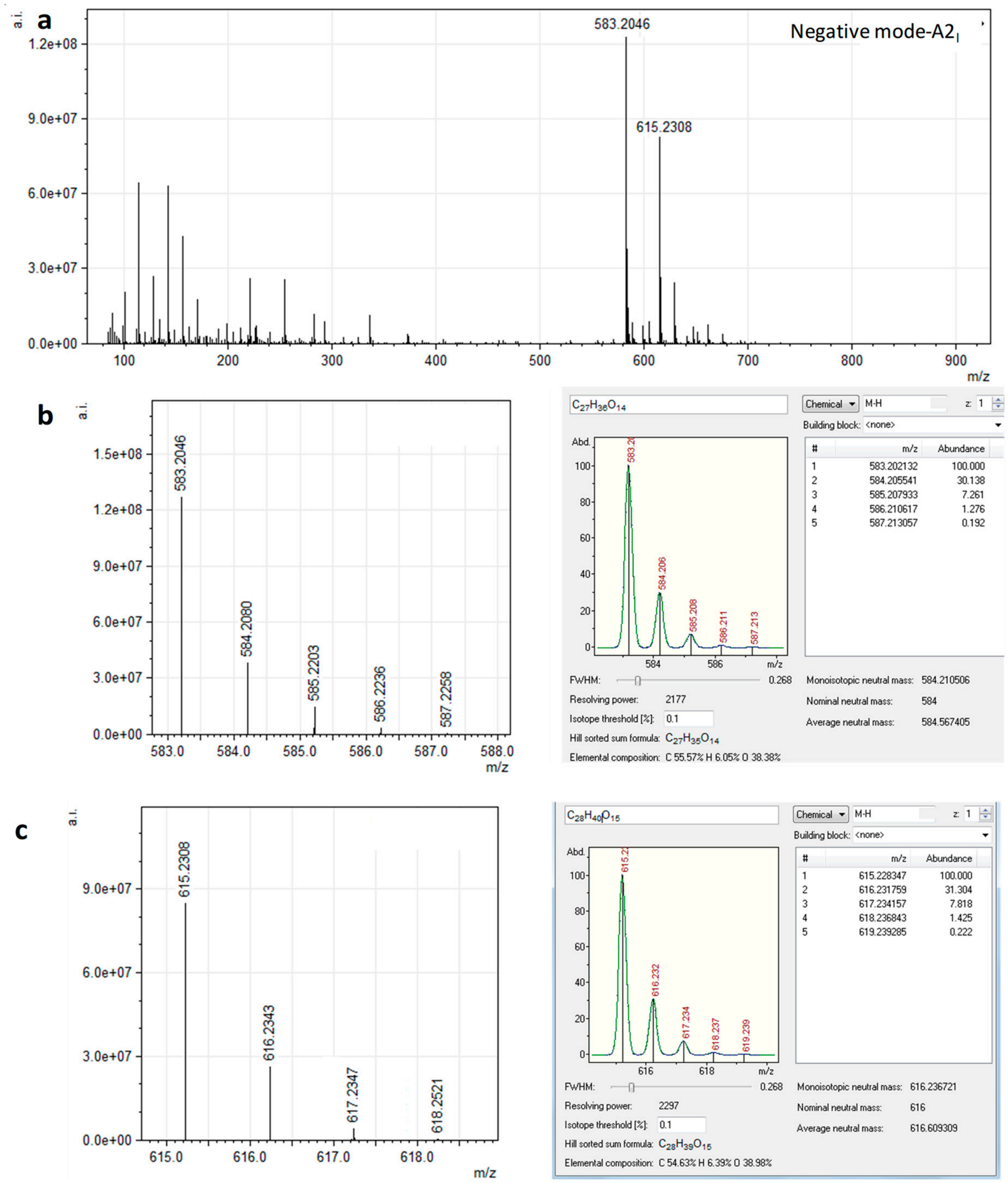
a
a.i.
Negative mode-A2
583.2046
615.2308
1.2e+08
9.0e+07
6.0e+07
3.0e+07
0.0e+00
100
200
300
400
500
600
700
800
900
m/z
b
a.i.
583.2046
584.2080
585.2203
586.2236
587.2258
1.5e+08
1.2e+08
9.0e+07
6.0e+07
3.0e+07
0.0e+00
583.0
584.0
585.0
586.0
587.0
588.0
m/z
C27H36O14
Chemical
M-H
z: 1
Building block: <none>
m/z Abundance
1 583.202132 100.000
2 584.205541 30.138
3 585.207933 7.261
4 586.210617 1.276
5 587.213057 0.192
Abd.
584.206
585.208
586.211
587.213
FWHM: 0.268
Resolving power: 2177
Isotope threshold [%]: 0.1
Hill sorted sum formula: C27H35O14
Elemental composition: C 55.57% H 6.05% O 38.38%
Monoisotopic neutral mass: 584.210506
Nominal neutral mass: 584
Average neutral mass: 584.567405
c
a.i.
615.2308
616.2343
617.2347
618.2521
9.0e+07
6.0e+07
3.0e+07
0.0e+00
615.0
616.0
617.0
618.0
m/z
C28H40O15
Chemical
M-H
z: 1
Building block: <none>
m/z Abundance
1 615.228347 100.000
2 616.231759 31.304
3 617.234157 7.818
4 618.236843 1.425
5 619.239285 0.222
Abd.
616.232
617.234
618.237
619.239
FWHM: 0.268
Resolving power: 2297
Isotope threshold [%]: 0.1
Hill sorted sum formula: C28H39O15
Elemental composition: C 54.63% H 6.39% O 38.98%
Monoisotopic neutral mass: 616.236721
Nominal neutral mass: 616
Average neutral mass: 616.609309

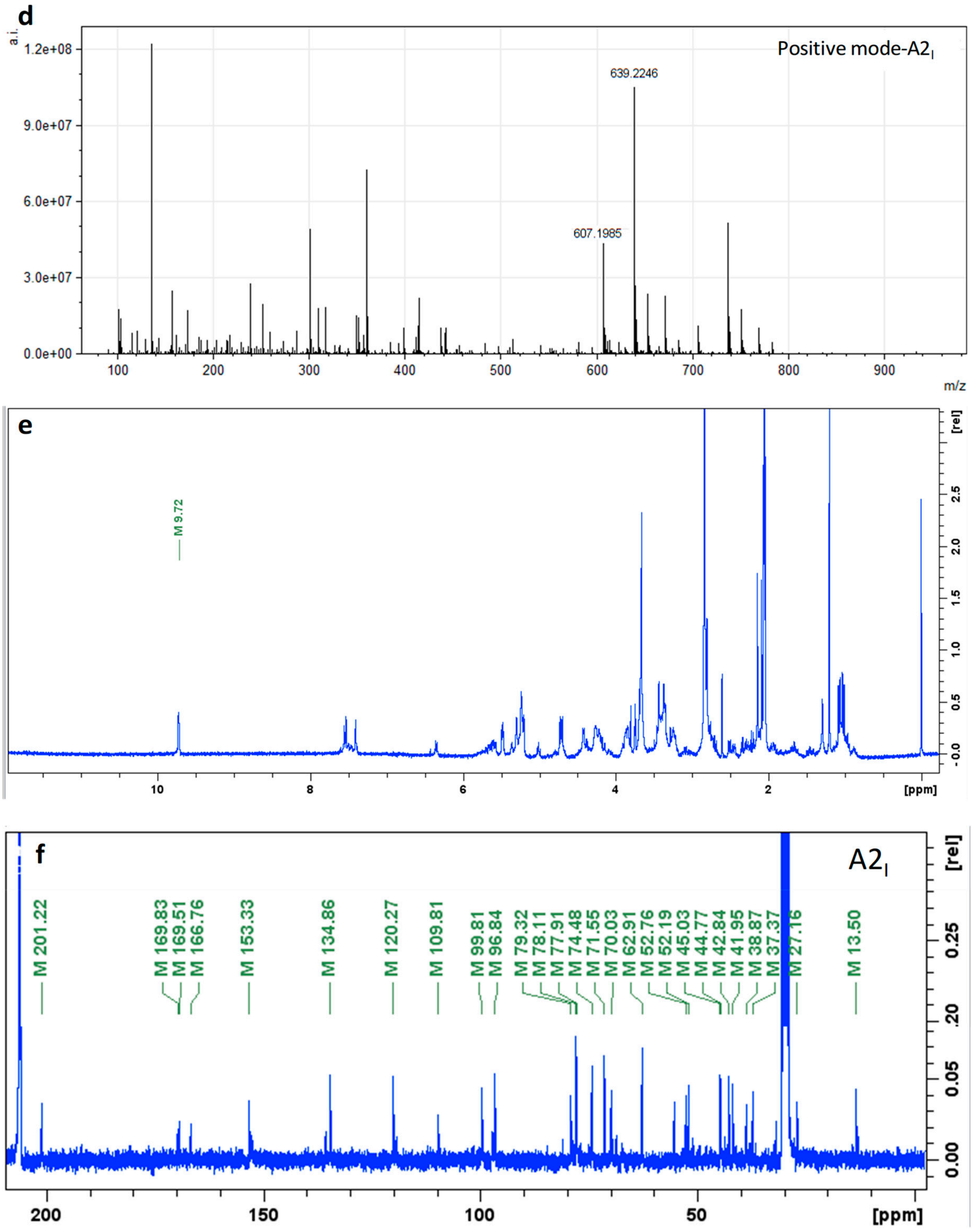

Figure S2. Fraction A2I: a) mass spectrum in negative mode, b) zoom mass spectrum in negative mode for *m*/*z* 583.2046, c) zoom mass spectrum in negative mode for *m*/*z* 615.2308, d) mass spectrum in positive mode, e) ^{1}H NMR spectrum (300 MHz) and, f) ^{13}C NMR spectrum (75 MHz).

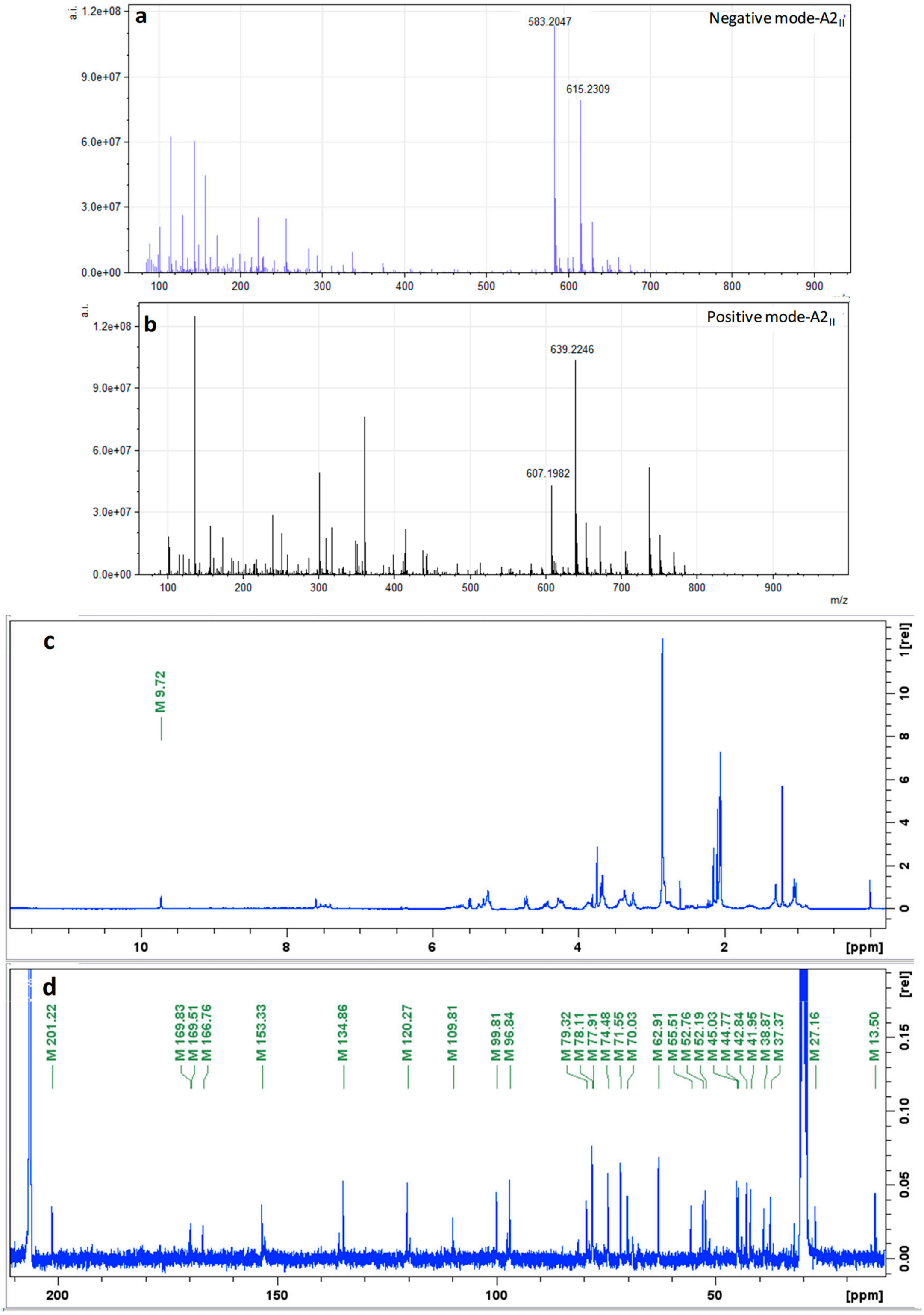

Figure S3. Fraction A2II: a) negative-ion mass spectrum, b) positive-ion mass spectrum, c) ^{1}H NMR spectrum (300 MHz) and, d) ^{13}C NMR spectrum (75 MHz).

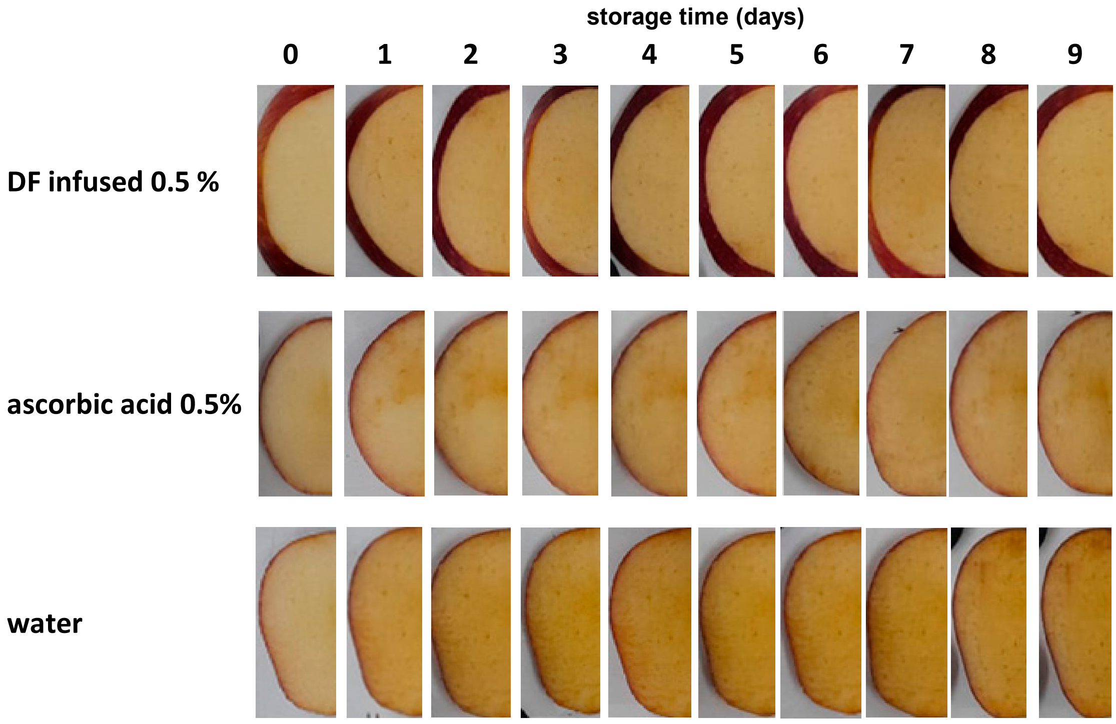

Figure S4: Representative images of apple slices treated with DF infusion extract (DF-5), ascorbic acid, or water during 9 days of storage in sealed polyethylene bags at 4 °C.

Table S1. Dose–response analysis of the DF-6 extract by the PPO–TLC assay. Halo diameters were measured on ImageJ-binarized images at twelve angles (15° intervals) through the spot centroid; values represent the mean ± standard deviation (SD) of these twelve diametral measurements per spot.

Dose [pixel]	mean diameter [pixel]	SD [pixel]	n	CV %
100	106	3.6	12	3.4
150	125.2	1.7	12	1.4
200	131	5.6	12	4.2
250	145.8	8.3	12	5.7
300	167.5	10	12	5.9
350	174.9	10.2	12	5.9
400	193.5	9.1	12	4.7
